# Ccp1-Ndc80 switch at the N terminus of CENP-T regulates kinetochore assembly

**DOI:** 10.1073/pnas.2104459118

**Published:** 2021-11-22

**Authors:** Qianhua Dong, Xue-lei Liu, Xiao-hui Wang, Yu Zhao, Yu-hang Chen, Fei Li

**Affiliations:** ^a^Department of Biology, New York University, New York, NY 10003-6688;; ^b^State Key Laboratory of Molecular Developmental Biology, Institute of Genetics and Developmental Biology, and Innovative Academy of Seed Design, Chinese Academy of Sciences, Beijing 100101, China;; ^c^College of Advanced Agricultural Sciences, University of Chinese Academy of Sciences, Beijing 100049, China;; ^d^Institute for Systems Genetics, Department of Biochemistry and Molecular Pharmacology, NYU Langone Health, New York, NY 10016

**Keywords:** kinetochore, centromere, CENP-A, phosphorylation, CENP-T

## Abstract

Kinetochores, a protein complex assembled on centromeres, mediate chromosome segregation. In most eukaryotes, centromeres are epigenetically specified by the histone H3 variant CENP-A. CENP-T, an inner kinetochore protein, serves as a platform for the assembly of the outer kinetochore Ndc80 complex during mitosis. How CENP-T is regulated through the cell cycle remains unclear. Ccp1 (counteracter of CENP-A loading protein 1) associates with centromeres during interphase but delocalizes from centromeres during mitosis. Here, we demonstrated that Ccp1 directly interacts with CENP-T. CENP-T is important for the association of Ccp1 with centromeres, whereas CENP-T centromeric localization depends on Mis16, a homolog of human RbAp48/46. We identified a Ccp1-interaction motif (CIM) at the N terminus of CENP-T, which is adjacent to the Ndc80 receptor motif. The CIM domain is required for Ccp1 centromeric localization, and the CIM domain–deleted mutant phenocopies *ccp1*Δ. The CIM domain can be phosphorylated by CDK1 (cyclin-dependent kinase 1). Phosphorylation of CIM weakens its interaction with Ccp1. Consistent with this, Ccp1 dissociates from centromeres through all stages of the cell cycle in the phosphomimetic mutant of the CIM domain, whereas in the phospho-null mutant of the domain, Ccp1 associates with centromeres during mitosis. We further show that the phospho-null mutant disrupts the positioning of the Ndc80 complex during mitosis, resulting in chromosome missegregation. This work suggests that competitive exclusion between Ccp1 and Ndc80 at the N terminus of CENP-T via phosphorylation ensures precise kinetochore assembly during mitosis and uncovers a previously unrecognized mechanism underlying kinetochore assembly through the cell cycle.

## Significance

Precise chromosome segregation relies on kinetochores. How kinetochores are precisely assembled on centromeres through the cell cycle remains poorly understood. Centromeres in most eukaryotes are epigenetically marked by nucleosomes containing the histone H3 variant, CENP-A. Here, we demonstrated that Ccp1, an anti–CENP-A loading factor, interacts with the N terminus of CENP-T to promote the assembly of the outer kinetochore Ndc80 complex. This work further suggests that competitive exclusion between Ccp1 and Ndc80 at the N terminus of CENP-T via phosphorylation ensures precise kinetochore assembly during mitosis. In addition, CENP-T is critical for Ccp1 centromeric localization, which in turn regulates CENP-A distribution. Our results reveal a previously unrecognized mechanism underlying kinetochore assembly through the cell cycle.

The precise inheritance of genetic information relies on the accurate segregation of chromosomes in mitosis and meiosis. Kinetochores are large protein complexes assembled on centromeres and play a crucial role in chromosome segregation. The kinetochore links the chromosome to microtubule polymers, drives the movement of chromosomes, and ensures correct microtubule–kinetochores attachment (1–3). The kinetochore assembly is thus tightly regulated. Yet, the mechanism by which kinetochores are precisely assembled through the cell cycle remains poorly understood.

The kinetochore comprises an outer region and an inner region. The outer kinetochore interacts with microtubules and is assembled on the platform of the inner kinetochore. The inner kinetochore consists of a complex of 14 to 16 subunits known as the constitutive centromere–associated network (CCAN) that is directly built on centromeric chromatin (4–6). In centromeres, the histone H3 variant, CENP-A, replaces the canonical histone H3 to form CENP-A–containing nucleosomes (7–9). Most eukaryotes contain large complex regional centromeres where CENP-A–containing nucleosomes are interspersed with canonical H3–containing nucleosomes (10–12). Regional centromeres are epigenetically specified by CENP-A (12–14). But how CENP-A– and histone H3–containing nucleosomes are balanced in centromeres remains unclear.

CENP-T, an integral component of CCAN, is also a histone fold–containing protein. CENP-T provides a platform for the assembly of the Ndc80 complex (Ndc80C), an essential outer kinetochore component, during mitosis (5, 15–18). Ndc80C acts as the interface between microtubules and kinetochores and mediates the microtubule attachments (19, 20). The long N terminus of CENP-T contains a conserved Ndc80 receptor motif. The motif forms an alpha-helix that directly interacts with the Spc24-Spc25 heterodimer in Ndc80C (15, 16). The motif can be phosphorylated by cyclin-dependent kinase 1 (CDK1) to stabilize the interaction between CENP-T and Ndc80C (16, 21–24). However, how CENP-T is regulated through the cell cycle to mediate the assembly of Ndc80C is still not well understood.

CENP-T has been shown to interact with three other histone fold–containing proteins, CENP-W, CENP-S, and CENP-X, to form the heterotetrameric nucleosome-like structure in vitro (25, 26). The CENP-T-W-S-X complex directly associates with centromeric DNA. The DNA binding activity of the complex is important for kinetochore formation (5, 25). Interestingly, the complex also directly associates with histone H3, not with CENP-A (5, 27), suggesting that CENP-T particles and the CENP-A nucleosome occupy different positions in centromeres. How the spatial relationship between the CENP-A nucleosome and CENP-T particles in centromeres is regulated remains unclear.

The fission yeast *Schizosaccharomyces pombe* contains large regional centromeres and is considered to be a model system for centromere study. The CENP-A homolog, Cnp1, is enriched in centromere cores, which are surrounded by pericentromeric heterochromatin (28–30). CENP-A^Cnp1^ nucleosomes nucleate kinetochore assembly. Mislocalization of CENP-A^Cnp1^ results in severe chromosome segregation defects in fission yeast (28, 31–34). Fission yeast also contains the CENP-T homolog, Cnp20, which associates with centromeres throughout the cell cycle. The same as in higher eukaryotes, CENP-T^Cnp20^ in *S. pombe* is essential for viability (35).

Recently, Ccp1, a nucleosome assembly protein (NAP) family protein, has been shown to play an important role in antagonizing the loading of CENP-A in fission yeast (36). Ccp1 forms a homodimer and is enriched at centromeres. Ccp1 acts as a key player in balancing CENP-A and histone H3 levels in the region (36). How Ccp1 regulates the CENP-A level in centromeres remains elusive. Interestingly, its centromere localization is cell cycle regulated. Ccp1 is dissociated from centromeres at the onset of mitosis and reassociates with centromeres at the end of mitosis (36, 37). The biological importance of the cell cycle–dependent interaction between Ccp1 and centromeres is unknown.

Here using mass spectrometry, we found that Ccp1 interacts directly with CENP-T^Cnp20^ in fission yeast. We further identified a conserved Ccp1-interaction motif (CIM) at the N terminus of CENP-T^Cnp20^, which is adjacent to the Ndc80 receptor motif. We demonstrated that CIM is important for Ccp1 localization. Furthermore, our data suggested that CDK1-mediated phosphorylation of the CIM motif at the onset of mitosis dissociates Ccp1 from CENP-T^Cnp20^, allowing proper positioning of Ndc80C. Ccp1 associates with centromeres during mitosis in the phospho-null mutant of the CIM domain, leading to mislocalization of Ndc80C and severe chromosome segregation defects. Our study uncovers a previously unrecognized mechanism regulating kinetochore organization in regional centromeres.

## Results

### CENP-T Interacts with Ccp1.

**Fig. 1.**
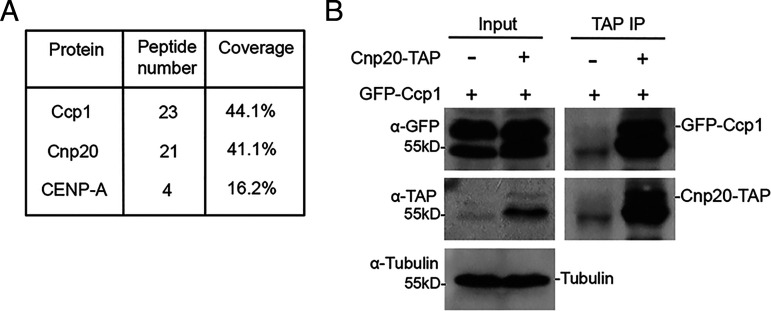
Ccp1 interacts with CENP-T. (*A*) CENP-T was identified by mass spectrometry analysis of purified Ccp1. The number of peptides identified and sequence coverage are shown. (*B*) Cell lysates from cells expressing GFP-Ccp1 and CENP-T^Cnp20^-TAP were immunoprecipitated with an antibody specific for TAP. Precipitated proteins were analyzed by Western blotting using indicated antibodies. Cells expressing GFP-Ccp1 only were used as a control.

We previously showed that Ccp1 localizes to the centromeric regions in all cell cycle stages except mitosis (36). In order to study the molecular mechanism underlying cell cycle–dependent localization of Ccp1, a functional tandem affinity purification (TAP)-tagged Ccp1 was used (36). We purified Ccp1-TAP by tandem affinity purification and subsequently used mass spectrometry to identify its interacting proteins. Our results identified two centromere-specific proteins, CENP-A^Cnp1^ and CENP-T^Cnp20^, associated with Ccp1 (Fig. 1*A* and *SI Appendix*, Fig. S1 and Dataset S1). Our previous TAP purification of CENP-A^Cnp1^ also revealed that CENP-A^Cnp1^ interacts with Ccp1 (36). The interaction of CENP-T^Cnp20^ and Ccp1 was further confirmed by coimmunoprecipitation (co-IP) (Fig. 1*B*). The C terminus of CENP-T^Cnp20^ contains a histone fold domain known to interact with CENP-W^New1^. Its N terminus is long, consisting of 383 amino acid (aa) residues. A conserved Ndc80 receptor motif from aa 70 to aa 87 was also identified in the N terminus (15).

### CENP-T Is Required for Ccp1 Centromere Localization.

**Fig. 2.**
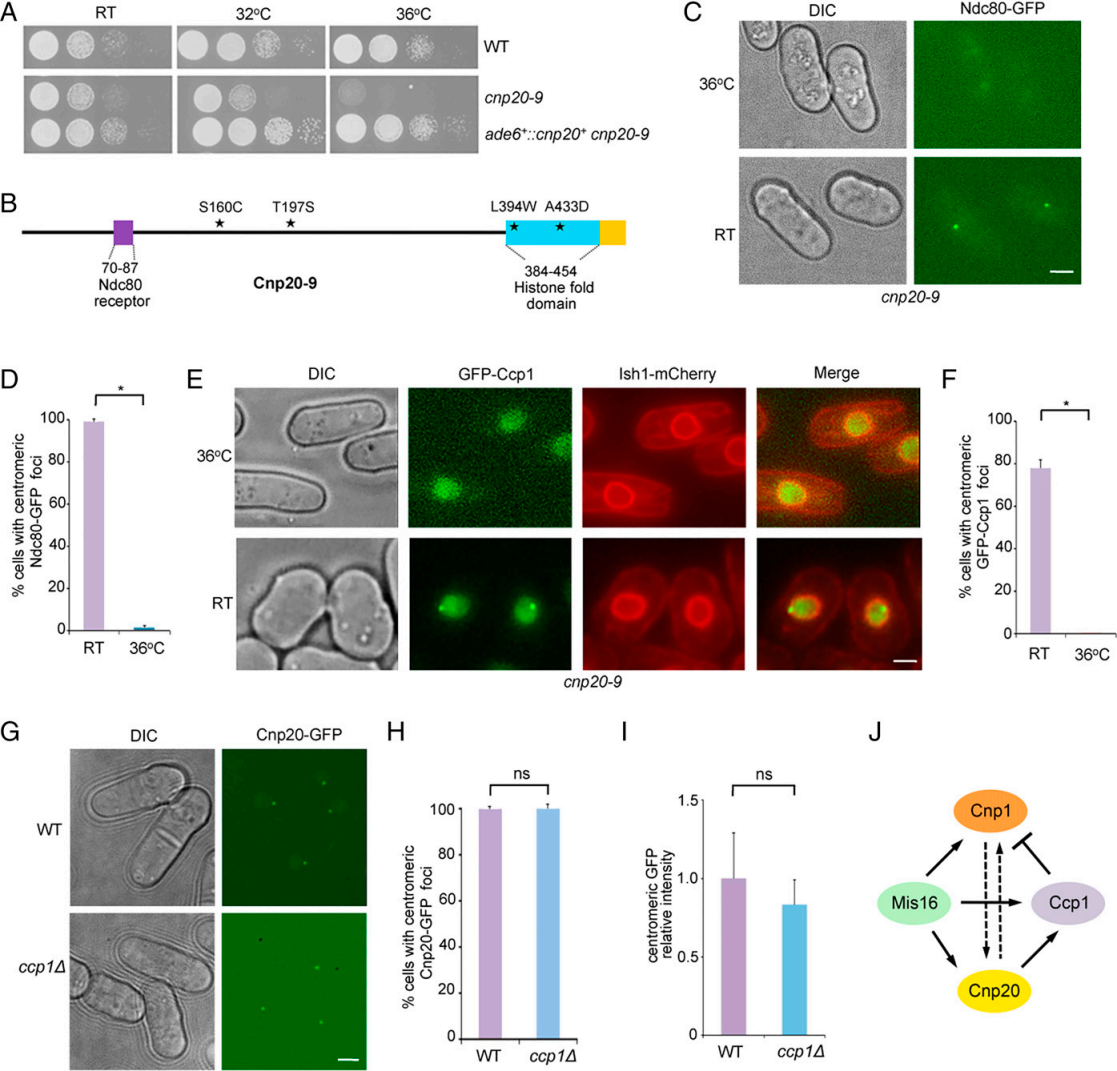
CENP-T is required for the centromeric localization of Ccp1. (*A*) Temperature sensitivity of *cnp20-9* strain. Serial dilutions of indicated cells were plated on YES media and incubated at indicated temperatures for 3 d. RT, room temperature. (*B*) Diagram showing mutations in Cnp20-9. ★, mutated amino acids; cyan, histone fold domain; purple, Ndc80 receptor motif. (*C*) The centromeric association of Ndc80-GFP is lost in *cnp20-9* at the restrictive temperature. The *cnp20-9* cells were incubated at 36 °C for 6 h. (*D*) Quantification of the percentage of cells showing centromeric localization of Ndc80-GFP. Experiments were performed in triplicate. At least 40 cells were scored in one single experiment. Error bars represent mean and SD. *P < 0.05. (*E*) The centromeric association of Ccp1 is abolished in *cnp20-9* at the restrictive temperature. The *cnp20-9* cells were incubated at 36 °C for 6 h. The nuclear envelope was visualized by Ish1-mCherry. (*F*) Quantification of the percentage of cells showing centromeric localization of GFP-Ccp1. Experiments were performed in triplicate. At least 40 cells were scored in one single experiment. Error bars represent mean and SD. *P < 0.05. (*G*) The centromeric association of CENP-T^Cnp20^ is not affected in the *ccp1*Δ mutant. (*H*) Quantification of the percentage of cells showing centromeric localization of CENP-T^Cnp20^-GFP. Experiments were performed in triplicate. At least 40 cells were scored in one single experiment. Error bars represent mean and SD. (*I*) Quantification of relative fluorescence intensity of centromeric CENP-T^Cnp20^-GFP. At least 50 cells were scored in one single experiment. Error bars represent mean and SD. ns, no significant differences. (Scale bars, 2 μm.) *n*, the number of cells counted. (*J*) Summary of localization dependency relationships between Ccp1, CENP-A^Cnp1^, CENP-T^Cnp20^, and Mis16. Dash arrow, partial localization dependency.

Like higher eukaryotes, CENP-T^Cnp20^ in fission yeast is essential for cell growth (35). To investigate the role of CENP-T^Cnp20^ in centromeres, we created a temperature-sensitive (*ts*) mutant of CENP-T^Cnp20^, *cnp20-9*, by random mutagenesis. The *cnp20-9* mutant grows normally at room temperature, but does not survive at 36 °C (Fig. 2*A*). Through sequencing, we identified four amino acid mutations in *cnp20-9*, including S160C and T197S in the N-terminal domain, and L394W and A433D in the histone fold domain (Fig. 2*B*). We also found that GFP-tagged Cnp20-9 is delocalized from the centromeres at the restrictive temperature (*SI Appendix*, Fig. S2). Consistent with the key role of CENP-T^Cnp20^ in the assembly of the Ndc80 complex, we found that the association of Ndc80-GFP with centromeres is lost in *cnp20-9* at the restrictive temperature (Fig. 2 *C* and *D*).

To determine the effect of *cnp20-9* on Ccp1 localization, we analyzed the *cnp20-9* strain carrying GFP-Ccp1. We found that GFP-Ccp1 was delocalized from centromeres at the restrictive temperature in *cnp20-9* at all stages of the cell cycle (Fig. 2 *E* and *F* and *SI Appendix*, Fig. S3), whereas the localization of GFP-Ccp1 at centromeres only has a mild reduction in the CENP-A *ts* mutant, *cnp1-1* (*SI Appendix*, Fig. S4). We next checked the distribution of CENP-T^Cnp20^-GFP in *ccp1*Δ and found that its centromere localization was unaffected in the mutant (Fig. 2 *G–I*).

It has been shown that, although CENP-T deposition is not dependent on CENP-A, the association of CENP-T with centromeres is reduced when CENP-A is knocked out in several species (5, 38–40). We examined the localization of CENP-T^Cnp20^-GFP in the *S. pombe cnp1-1* mutant and showed that CENP-T^Cnp20^ at centromeres is also partially decreased at 36 °C (*SI Appendix*, Fig. S5). In addition, we found that CENP-A^Cnp1^-GFP partially reduced its centromere localization in *cnp20-9* at the restrictive temperature (*SI Appendix*, Fig. S6). We have previously shown that Ccp1 dissociates from centromeres in the *mis16* mutant (36). We next examined CENP-T^Cnp20^-GFP distribution in the *mis16-53* mutant. We found that the association of CENP-T^Cnp20^ with centromeres is largely lost at the restrictive temperature in the mutant background (*SI Appendix*, Fig. S7). It is thus likely that Mis16 may regulate Ccp1 localization via CENP-T^Cnp20^. It has been shown that Mis16 centromeric localization is not affected by the *cnp1-1* mutant at the restrictive temperature (41), consistent with our observation that Ccp1 dissociates from centromeres in the *mis16* mutant but still can stay at centromeres in *cnp1-1*. Fig. 2*J* shows a summary of localization dependency relationships between Ccp1, CENP-A^Cnp1^, CENP-T^Cnp20^, and Mis16. Together, our data indicate that CENP-T^Cnp20^ is required for Ccp1 centromere localization.

### Ccp1 Interacts with the CIM Domain at the N Terminus of CENP-T.

**Fig. 3.**
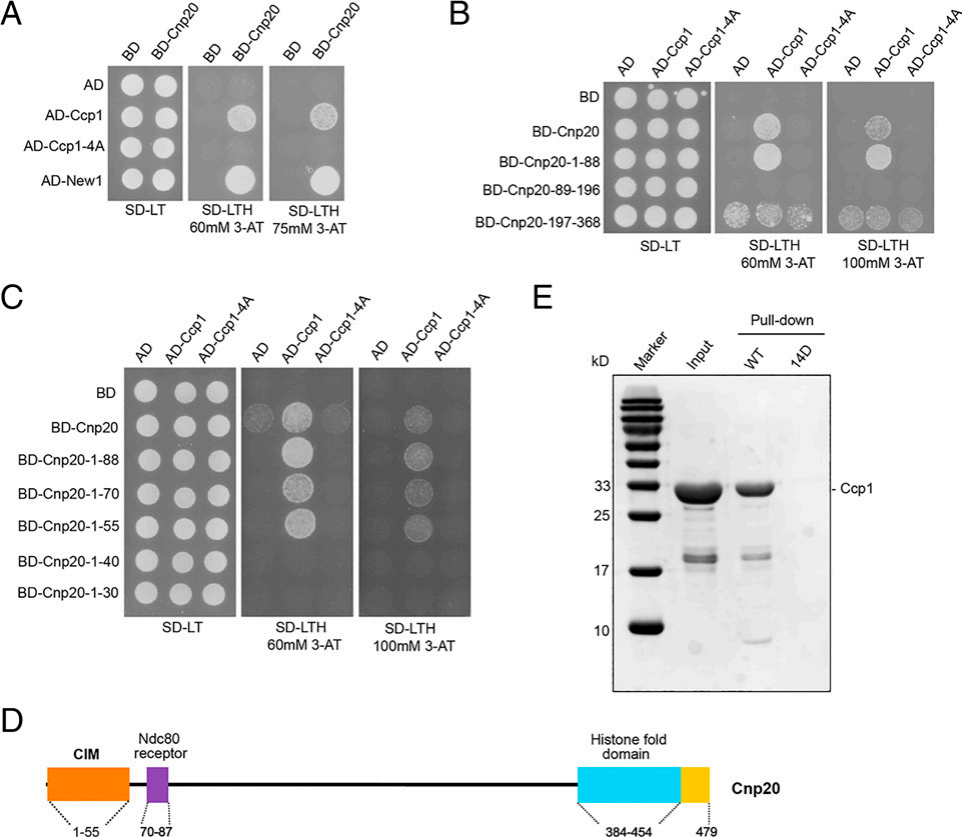
The N terminus of CENP-T contains a conserved CIM. (*A*) Yeast two-hybrid (Y2H) analysis confirmed the interaction between Ccp1 and CENP-T^Cnp20^ and also showed that the interaction is lost in the dimeric mutant of *ccp1-4A*. *S. cerevisiae* strains carrying the indicated plasmid combinations were spotted onto synthetic defined (SD) minimal medium plates lacking either Leu and Trp (SD −LT) or Leu, Trp, and His (SD −LTH) supplemented with either 60 mM 3-amino-1,2,4-triazole (3-AT) or 75 mM 3-AT, and grew at 30 °C. AD, activation domain; BD, binding domain. (*B*) Y2H assays indicated that Ccp1 associates with the first 88 amino acids at the CENP-T^Cnp20^. (*C*) The first 55 amino acids of CENP-T^Cnp20^ were identified by Y2H as a minimum Ccp1-interacting motif. (*D*) A schematic diagram of the domain structure of CENP-T^Cnp20^. Orange, the CIM domain; cyan, histone fold domain; purple, Ndc80 receptor motif. (*E*) In vitro pull-down assay demonstrates that the first 55 amino acids of CENP-T^Cnp20^ interact with Ccp1, but the interaction of the phosphomimetic 14D mutant of the region (14D) with Ccp1 is disrupted. Wild-type and phosphomimetic 14D mutant peptides derived from Cnp20^1-55^, and purified full-length Ccp1 were used.

To further confirm that Ccp1 interacts with CENP-T^Cnp20^, the yeast two-hybrid system was performed in a heterologous system *Saccharomyces cerevisiae*. The CENP-W^New1^ known to have strong interaction with CENP-T^Cnp20^ was used as a control. Our yeast two-hybrid assays confirmed the interaction between Ccp1 and CENP-T^Cnp20^ (Fig. 3*A*). Our previous study indicated that Ccp1 forms a homodimer, which is necessary for the centromeric localization of Ccp1 (36). The yeast two-hybrid assays showed that the Ccp1 homodimer mutant, Ccp1-4A, was unable to interact with CENP-T^Cnp20^ (Fig. 3*A* and *SI Appendix*, Fig. S8), indicating that the dimeric structure is vital for its interaction with CENP-T^Cnp20^.

In order to identify the interacting domain of CENP-T^Cnp20^ with Ccp1, we constructed the N-terminal and C-terminal domains of CENP-T^Cnp20^: Cnp20-N (aa 1 to 368) and Cnp20-C (aa 369 to 479), respectively. Our yeast two-hybrid assays indicated that Ccp1 interacts with the N-terminal domain but not with the C terminus (*SI Appendix*, Fig. S9). To narrow down the Ccp1-interacting region of CENP-T^Cnp20^, we constructed a series of N-terminal fragments of CENP-T^Cnp20^, including aa 1 to 88, aa 89 to 196, and aa 197 to 368, and conducted yeast two-hybrid assays using Ccp1 as a bait. We found that Ccp1 associates with aa 1 to 88 (Fig. 3*B*). Further yeast two-hybrid analysis using partial deletion of the first 88 amino acids revealed that the first 55 amino acids interacts with Ccp1 at a similar level as the full-length CENP-T^Cnp20^, whereas fragments aa 16 to 55 and aa 1 to 44 showed much weaker interaction (Fig. 3*C* and *SI Appendix*, Fig. S10). Our results indicate that the first 55 amino acids of CENP-T^Cnp20^ are the minimal interaction domain with Ccp1, which we named the Ccp1-interacting motif (CIM). The CIM domain lies close but does not overlap with the Ndc80 receptor motif (Fig. 3*D*). Our sequence analysis also identified multiple residues within the CIM domain that are conserved in CENP-T orthologs in *Gallus gallus*, *Homo sapiens*, and *Mus musculus*, as well as *S. cerevisiae* (*SI Appendix*, Fig. S11).

To further investigate how the CIM domain physically interacts with Ccp1, we performed in vitro pull-down assays using a peptide derived from Cnp20^1-56^ and purified full-length Ccp1. We found that Cnp20^1-56^ was able to pull down Ccp1 in vitro, indicating that the CIM domain indeed directly interacts with Ccp1 (Fig. 3*E*). We also performed in vitro pull-down assays using three peptides derived from Cnp20^1-25^, Cnp20^15-40^, and Cnp20^31-56^, respectively. We found that both Cnp20^15-40^ and Cnp20^31-56^ were able to pull down Ccp1 in vitro, and their interactions with Ccp1-4A are reduced (*SI Appendix*, Fig. S12).

### The CIM Domain of CENP-T Is Important for Ccp1 Localization.

**Fig. 4.**
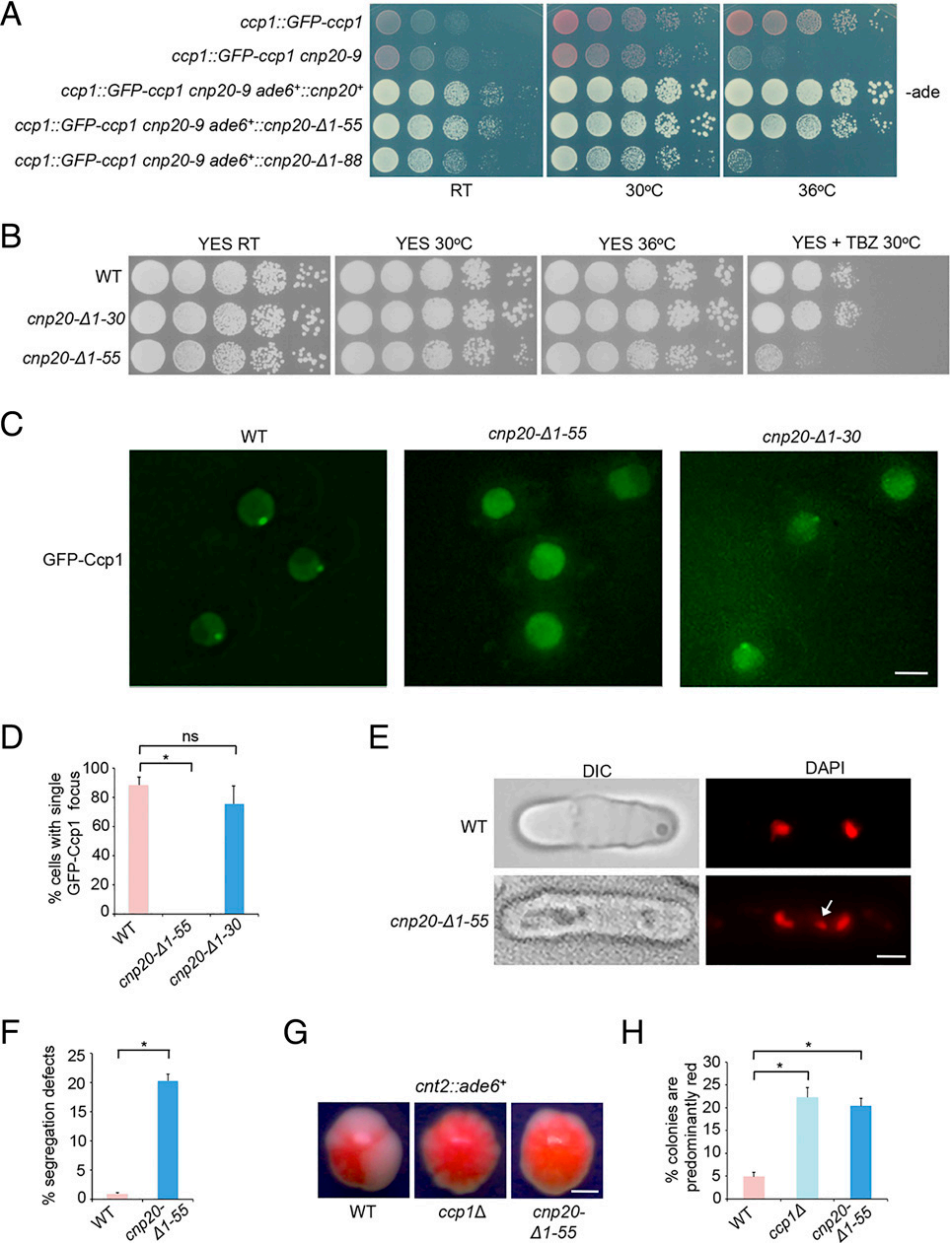
The CIM domain–deleted CENP-T phenocopies the *ccp1*Δ mutant. (*A*) The *cnp20-9* mutant carrying the CIM domain–deleted CENP-T^Cnp20^ (*cnp20-*Δ*1–55*) was viable at restrictive temperature, whereas deletion of the first 88 amino acids of CENP-T^Cnp20^ results in cell death. Cells grew on the rich YES medium with low adenine (-ade) for 3 d at the indicated temperatures. RT, room temperature. (*B*) The CIM domain–deleted CENP-T^Cnp20^ is sensitive to TBZ. Cells grew on the rich YES medium in the absence or presence (15 μg/mL) of TBZ at indicated temperatures for 3 to 5 d. (*C*) GFP-Ccp1 dissociates from centromeres in *cnp20-*Δ*1–55*. Exponentially growing cells at 30 °C were used. (Scale bar, 2 μm.) (*D*) Quantification of the percentage of cells showing centromeric localization of GFP-Ccp1. Experiments were performed in triplicate. At least 40 cells were scored in one single experiment. Error bars represent mean and SD. *P < 0.05. ns, no significant differences. (*E*) The *cnp20-*Δ*1–55* mutant cells display chromosome missegregation defects during mitosis, as shown by DAPI staining. Lagging chromosomes (white arrow) were frequently observed in *cnp20-*Δ*1–55*. (Scale bar, 2 μm.) (*F*) The percentage of mitotic cells showing lagging chromosomes. Experiments were performed in triplicate. At least 40 cells were scored in one single experiment. Error bars represent mean and SD. *P < 0.05. (*G*) Colony color sectoring morphology analysis of indicated cells carrying *cnt2::ade6^+^.* Both *cnp20-*Δ*1–55* and *ccp1*Δ displayed fewer sectors than WT. (Scale bar, 0.5 mm.) (*H*) Quantification of the percentage of predominantly red colonies. Experiments were performed in triplicate. At least 40 colonies were scored in one single experiment. Error bars represent mean and SD. *P < 0.05.

To determine the role of the CIM domain of CENP-T^Cnp20^ in Ccp1 localization, a CIM domain–deleted version of *cnp20*^+^ (*cnp20*-Δ*1–55*) was constructed under the control of its endogenous promoter and inserted at the *ade6*^+^ locus in the *cnp20-9* mutant background. As a control, the full-length *cnp20*^+^ and the first 88 amino acid–deleted *cnp20* (*cnp20*-Δ*1–88*) were also inserted in *ade6*^+^ in the same manner. The full-length *cnp20*^+^ can completely rescue the growth defect of *cnp20-9* at the restrictive temperature. On the other hand, cells carrying c*np20*-Δ*1–88* grow well at room temperature, but not at 36 °C, the same as the *cnp20-9* mutant alone (Fig. 4*A*). We further found that the deletion of the first 55 amino acids can also rescue *cnp20-9*, but has a slight growth defect (Fig. 4*A*). These results indicated that the CIM domain is not essential for cell viability, similar to the *ccp1*Δ mutant shown previously, whereas deletion of the Ndc80 receptor motif is lethal.

We next deleted the CIM domain of the endogenous *cnp20*^+^ (*cnp20*-Δ*CIM*) by CRISPR-Cas9. As a control, we also created a strain in which the first 30 amino acids in CENP-T^Cnp20^ (*cnp20*-Δ*1–30*) were deleted (Fig. 4*B*). To determine how the CIM domain affects Ccp1 localization, we examined the GFP-Ccp1 pattern in these strains. In the *cnp20*-Δ*1–30* mutant, we found that GFP-Ccp1 is enriched in centromeres during interphase similar to wild type (WT), though the GFP signal is weaker (Fig. 4 *C* and *D*). But GFP-Ccp1 is completely disassociated from centromeric regions in *cnp20*-Δ*CIM* at all stages of the cell cycle (Fig. 4 *C* and *D* and *SI Appendix*, Fig. S13). We concluded that the CIM domain is required for Ccp1 centromeric localization. Our data suggest that CENP-T regulates the recruitments of Ccp1 to centromeres via its CIM domain.

### *cnp20-*Δ*CIM* Phenocopies the *ccp1*Δ Mutant.

Our previous study had shown that the *ccp1*Δ mutant is sensitive to the microtubule destabilizing drug, thiabendazole (TBZ) due to chromosome segregation defects (36). To determine whether TBZ has a similar effect to *cnp20-*Δ*CIM*, we analyzed the growth of the mutant in rich media with TBZ as well as *cnp20*-Δ*1–30*. Our growth assays indicated that while the deletion of the first 30 amino acids has no significant growth defect, *cnp20-*Δ*CIM* displayed a TBZ-sensitive phenotype (Fig. 4*B*). Consistent with the results, our DAPI staining showed that more than 16% of *cnp20-*Δ*CIM* cells contain lagging chromosomes during mitosis (Fig. 4 *E* and *F*).

The epigenetic state of *S. pombe* centromeres is manifested by position effect variegation (PEV). Reporter genes inserted within the centromeric core region are silenced stochastically. The silencing state correlates with the CENP-A nucleosome occupancy in centromeres (42, 43). Deletion of *ccp1*^+^ results in increased silencing in centromere cores (37). To investigate the effect of *cnp20-*Δ*CIM* on PEV in centromeres, we crossed the mutant into the background with *ade6*^+^ in the centromere core. Silencing of *ade6*^+^ results in red colony color in the adenine-limiting media while its expression leads to white colonies. Wild-type cells carrying the reporter in the media thus display red/white color sectoring morphology due to PEV in centromeres; but colonies of *ccp1*Δ are mostly red due to increased silencing in the region (37). We found that single colonies of *cnp20-*Δ*CIM* also contain fewer white sectors than those of WT, and are predominantly red (Fig. 4 *G* and *H*). In addition, we found that similar to *ccp1*Δ (36), ∼20% of *cnp20-*Δ*CIM* mutant cells with a single fluorescence focus have disproportionately brighter CENP-A^cnp1^-GFP signal (*SI Appendix*, Fig. S14), suggesting that the level of CENP-A^cnp1^ in centromeres substantially increase in these cells. Together, these results indicate that *cnp20-*Δ*CIM* phenocopies the *ccp1*Δ mutant, consistent with the idea that CENP-T^Cnp20^ recruits Ccp1 through its CIM domain to centromeres during interphase.

### The Interaction between CENP-T and Ccp1 Is Disrupted in the *cnp20-14D* Mutant.

We have demonstrated that CENP-T^Cnp20^ interacts with and recruits Ccp1 to centromeres through its CIM domain. CENP-T^Cnp20^ in *S. pombe* is an inner kinetochore protein known to associate with centromeric regions at all stages of the cell cycle (44), whereas Ccp1 is disassociated from centromeres during mitosis. The mechanism underlying the disassociation of Ccp1 from centromeres at the onset of mitosis is not known, nor is its biological significance.

**Fig. 5.**
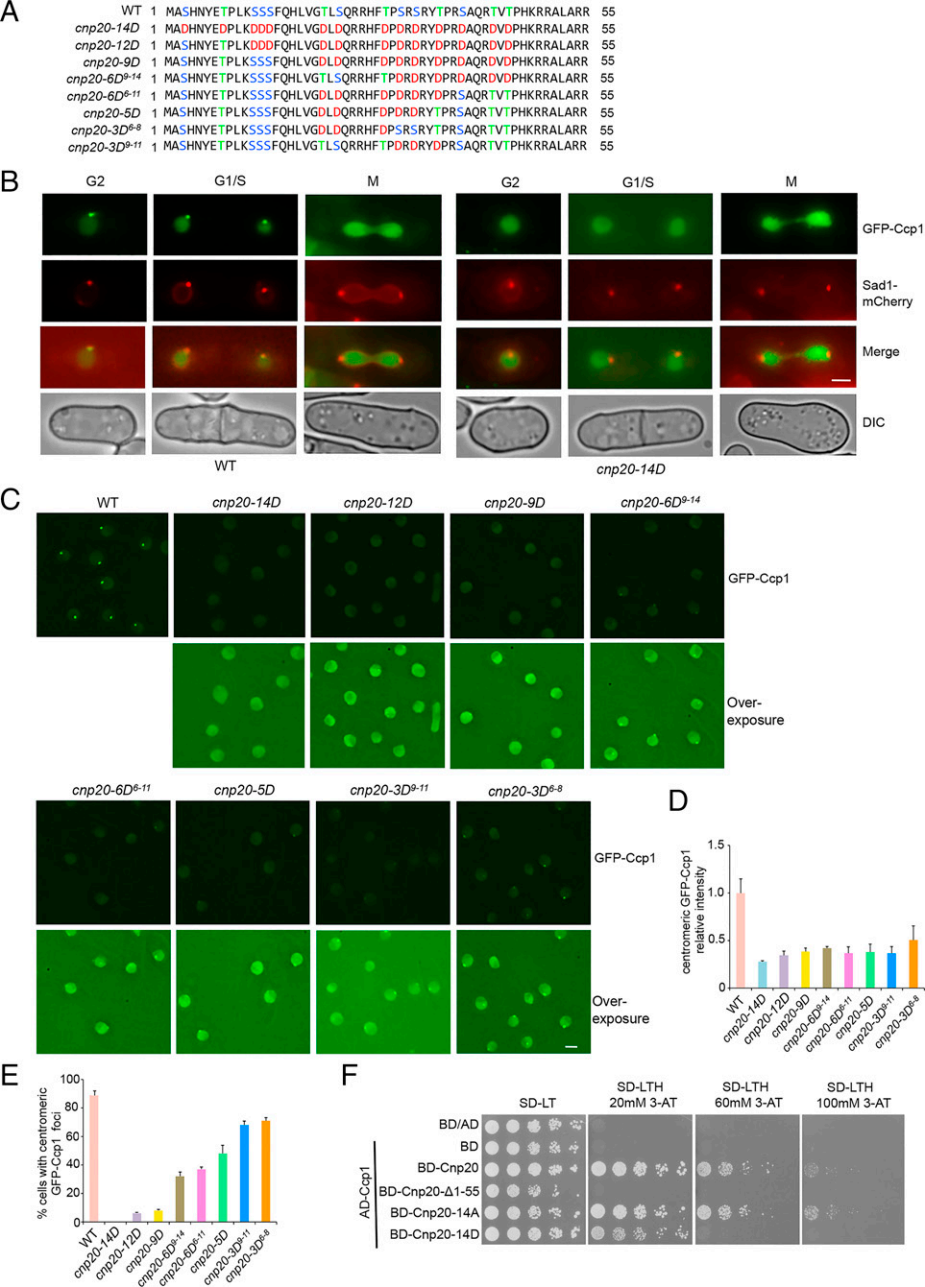
The association of Ccp1 with centromeres is abolished in the *cnp20-14D* mutant. (*A*) Schematic representation of the wild-type CIM domain and its phosphomimetic mutants. (*B*) Ccp1 dissociates from centromeres through all stages of the cell cycle in the *cnp20-14D* mutant. Sad1-mCherry was used as an SPB marker. (*C*) The distribution pattern of GFP-Ccp1 in indicated phosphomimetic mutants. Wild-type cells carrying GFP-Ccp1 were used as a control. (*D*) Quantification of relative fluorescence intensity of centromeric GFP-Ccp1 in the indicated cells. At least 40 cells were scored in one single experiment. Error bars represent mean and SD. (*E*) Quantification of the percentage of cells showing centromeric localization of GFP-Ccp1 in the indicated cells. Experiments were performed in triplicate. At least 40 cells were scored in one single experiment. Error bars represent mean and SD. (*F*) The Y2H analysis indicated that the interaction of Ccp1 with phosphomimetic Cnp20-14D mutant was weakened. (Scale bars, 2 μm.)

Previous studies have shown that CENP-T was phosphorylated at multiple sites at its N terminus, including the conserved site on S74 within the Ndc80 receptor motif in *S. cerevisiae*, and T72 and S88 in *G. gallus* (16, 21, 22). Phosphorylation of CENP-T plays an important role in the recruitment of Ndc80C during mitosis (21–24). We identified 14 serine and threonine residues in the CIM domain of CENP-T^Cnp20^ that can be potentially phosphorylated (Fig. 5*A*). We reasoned that the phosphorylation of the CIM domain may regulate the dissociation of Ccp1 from the centromeric region during mitosis. To test this idea, we mutated all serine and threonine residues in the CIM domain to phosphomimetic aspartic acid (*cnp20*-*14D*) at the endogenous sites by CRISPR-Cas9 (Fig. 5*A*). We found that, unlike in WT, the GFP-Ccp1 was completely delocalized from centromeres at all phases of the cell cycle (Fig. 5 *B* and *C*). These data suggest that phosphorylation of the CIM domain leads to disassociation of Ccp1 from centromeres.

In order to find the minimal phosphorylation sites that contribute to the centromeric positioning of Ccp1, we mutated a series of serine and threonine residues to aspartic acid, including *cnp20*-*12D*, *cnp20*-*9D*, *cnp20*-*6D^9-14^*, *cnp20*-*6D^6-11^*, *cnp20*-*5D*, *cnp20*-*3D^6-8^*, and *cnp20*-*3D^9-11^* at its endogenous locus (Fig. 5*A*). We examined the localization of GFP-Ccp1 using the same approach. We found that GFP-Ccp1 is still associated with centromeres in all these mutants, although at different degrees (Fig. 5 *C–E*). Ccp1 centromeric distribution is the least affected in *cnp20*-*3D^6-8^* and *cnp20*-*3D^9-11^*, whereas most *cnp20*-*12D* cells display delocalized GFP-Ccp1, although not as severely as in *cnp20*-*14D*. These results indicated that all predicted phosphorylation sites within the CIM domain of CENP-T^Cnp20^ are involved in the association of Ccp1 with centromeres.

To investigate how phosphorylation of the CIM domain affects the interaction between Ccp1 and CENP-T^Cnp20^, we conducted yeast two-hybrid assays with Cnp20-14D and Ccp1. We found that the interaction between Cnp20-14D and Ccp1 was dramatically reduced (Fig. 5*F*). This is further confirmed by our in vitro binding assays (Fig. 3*E*). These data suggest that phosphorylation of the 14 serine and threonine residues within the CIM domain disrupts the interaction between CENP-T and Ccp1, which explains the Ccp1 delocalization phenotype in *cnp20*-*14D*. These results also suggest that a combination of phosphorylation events within the CIM domain leads to the disassociation of Ccp1 from CENP-T^Cnp20^ during mitosis.

### Ccp1 Associates with Centromeres during Mitosis in the *cnp20-14A* Mutant.

**Fig. 6.**
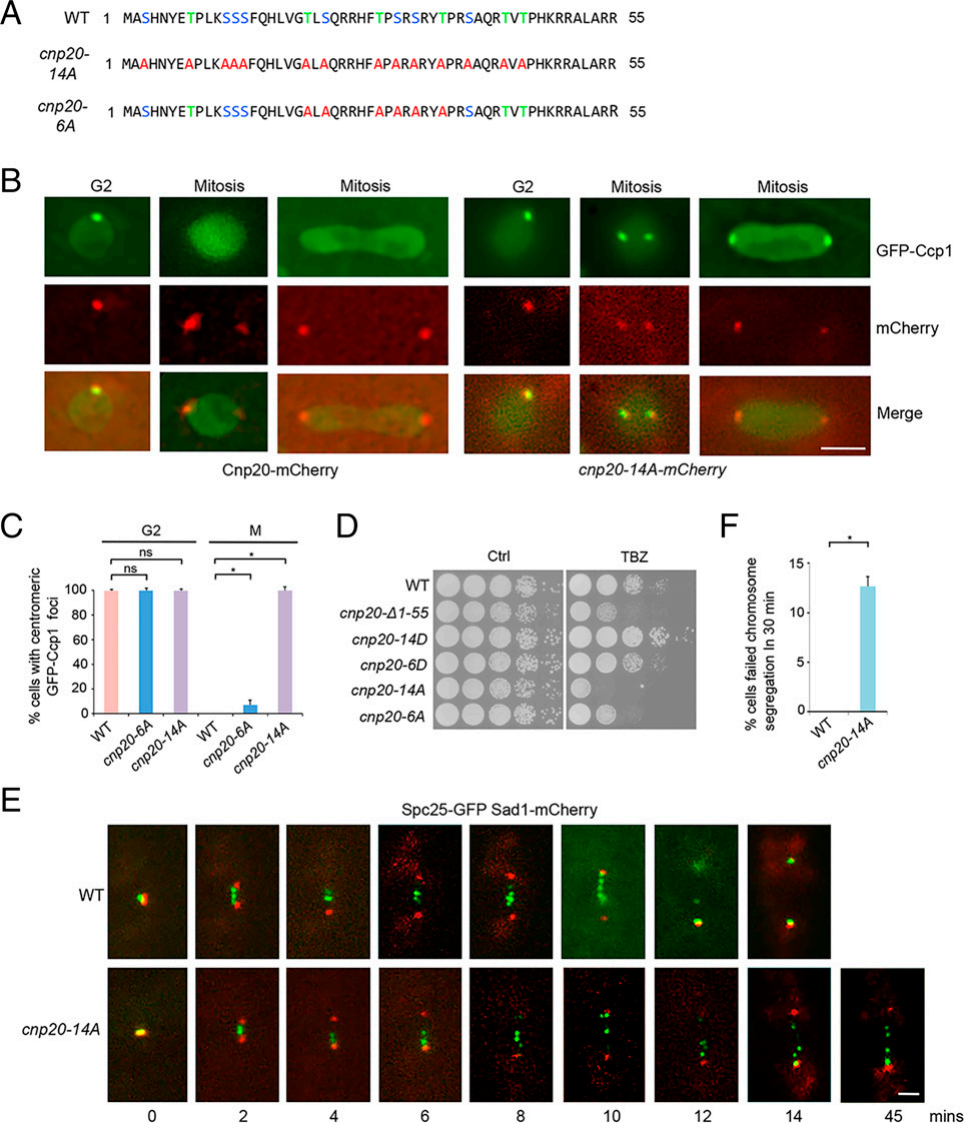
Ccp1 associates with centromeres during mitosis in the *cnp20-14A* mutant. (*A*) Schematic representation of the WT CIM domain and its phospho-null mutants. (*B*) GFP-Ccp1 associates with centromeres during mitosis in the *cnp20-14A* mutant. Cnp20- or Cnp20-14A-mCherry was used as a centromere marker. (Scale bar, 2 μm.) (*C*) Quantification of the percentage of indicated cells showing centromeric localization of GFP-Ccp1 in mitosis (M) and G2. Experiments were performed in triplicate. At least 40 cells were scored in one single experiment. Error bars represent mean and SD. *P < 0.05. ns, no significant differences. (*D*) TBZ sensitivity of phospho-null or phosphomimetic CIM mutants. Cells grew on the rich YES medium in the absence or presence (15 μg/mL) of TBZ at 30 °C for 3 to 5 d. (*E*) Time-lapse microscopy of Spc25-GFP and Sad1-mCherry in WT (*Top*) and *cnp20-14A* (*Bottom*). Time was measured from the separation of SPB (0 min). Sad1-mCherry was used as an SPB marker. (Scale bar, 1 μm.) (*F*) Quantification of the percentage of indicated cells that failed to complete chromosome segregation in 30 min. Experiments were performed in triplicate. At least 40 cells were scored in one single experiment. Error bars represent mean and SD. *P < 0.05.

We next investigated how the dephosphorylation of the CIM domain in CENP-T^Cnp20^ affects Ccp1 localization. We replaced all of the 14 serine and threonine residues with phospho-null alanine in the CIM domain of CENP-T^Cnp20^ at its endogenous site (*cnp20*-*14A*) and examined the distribution pattern of GFP-Ccp1 throughout the cell cycle in the mutant background. As a control, we also created a mutant in which 6 serine and threonine residues within CIM were replaced by alanine (*cnp20*-*6A*) (Fig. 6*A*). GFP-Ccp1 in WT is delocalized from centromeres during mitosis. In contrast, we found that GFP-Ccp1 in all *cnp20*-*14A* mutant cells remains associated with centromeres during all the stages of the cell cycle (Fig. 6 *B* and *C*). We also observed centromeric localization of GFP-Ccp1 in *cnp20*-*6A* cells, although the GFP signal is very weak (*SI Appendix*, Fig. S15). These data indicate that dephosphorylation of the CIM of CENP-T^Cnp20^ results in a tight association of Ccp1 with centromeres. Consistent with this idea, our yeast two-hybrid assays demonstrated that, unlike Cnp20-14D, Cnp20-14A showed a strong interaction with Ccp1 (Fig. 5*F*). Together, our results suggest that the CIM domain of CENP-T^Cnp20^ is phosphorylated at the onset of mitosis leading to dissociation of Ccp1 from centromeres, while the dephosphorylation of the CIM domain at the end of mitosis allows Ccp1 to reassociate with centromeres and remain stably bound throughout interphase.

### The *cnp20-14A* Mutant Disrupts the Positioning of the Ndc80 Complex during Mitosis.

To determine the effect of *cnp20*-*14A* on chromosome segregation, we analyzed the mutant using TBZ growth assays. We found that *cnp20*-*14A* is highly sensitive to TBZ, even more strongly than the *cnp20*-Δ*CIM* mutant (Fig. 6*D* and *SI Appendix*, Fig. S16), indicating that *cnp20*-*14A* leads to severe chromosome segregation defects. We further utilized time-lapse microscopy to examine chromosome segregation in *cnp20*-*14A*. The mutant cells expressing the GFP-tagged Spc25, a subunit of Ndc80C and the Sad1-mCherry (spindle pole body [SPB] marker), were analyzed. After the onset of mitosis, kinetochores oscillate between the two SPBs and segregate toward the SPBs during anaphase. Wild-type cells complete the segregation within 14 min (Fig. 6*E*). But most *cnp20*-*14A* cells display mitotic delay, and more than 12% of mutant cells failed to complete chromosome segregation within 30 min (Fig. 6 *E and F*).

**Fig. 7.**
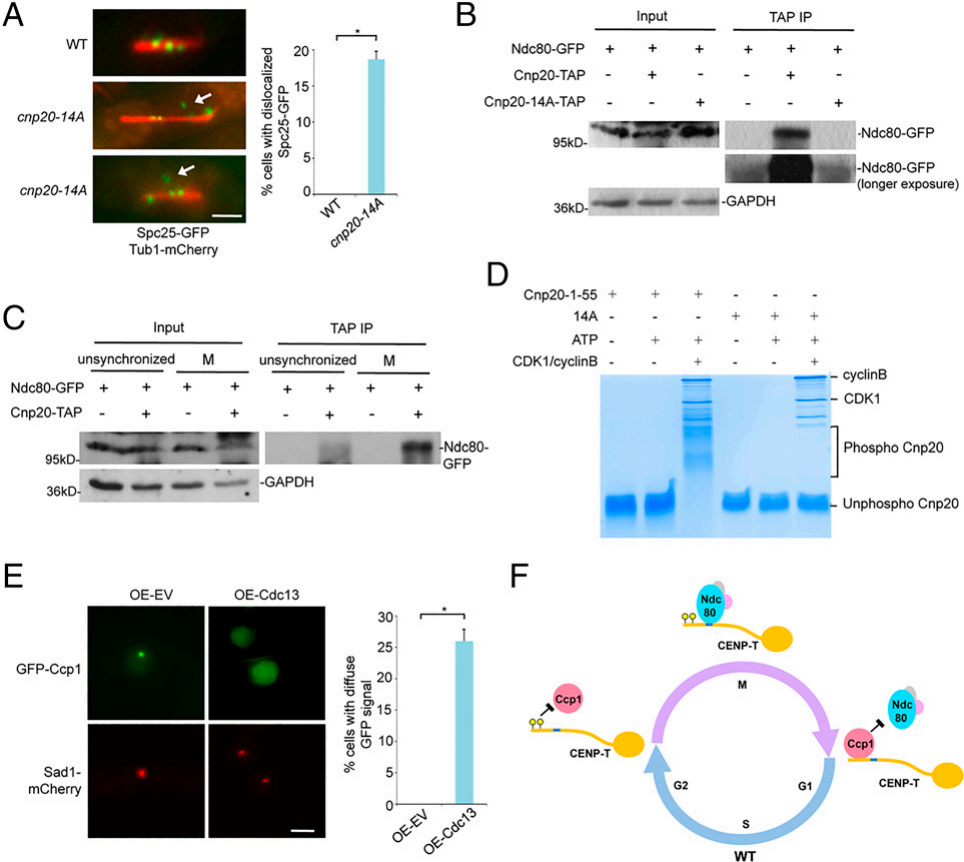
The interaction of Ndc80-CENP-T is abolished in the phospho-null CIM mutant and CDK1 phosphorylates the CIM domain. (*A*) Spc25-GFP localization in the *cnp20-14A* mutant is aberrant (white arrow) during mitosis. Tub1-mCherry was used as a microtubule marker. *Right*: Quantification of the percentage of indicated cells with mislocalized Spc25-GFP. (Scale bar, 2 μm.) (*B*) Lysates from indicated cells synchronized at metaphase using *nda3-*KM311 were immunoprecipitated with an antibody specific for TAP. Precipitated proteins were analyzed by Western blotting using a GFP antibody. GAPDH was used as a loading control. (*C*) Cell lysates from cells expressing Ndc80-GFP and CENP-T^Cnp20^-TAP either synchronized in mitosis or unsynchronized were immunoprecipitated with an antibody specific for TAP. Precipitated proteins were analyzed by Western blotting using indicated GFP antibody. Cells expressing Ndc80-GFP were used only as a control. GAPDH was used as a loading control. (*D*) In vitro kinase assays were performed using human recombinant CDK1-cyclin B and a synthesized peptide derived from CENP-T^Cnp20^ 1 to 55. The CENP-T^Cnp20^ 1 to 55-14A peptide (14A) was used as a control. Samples were analyzed by Phos-tag PAGE. (*E*) Constitutive overexpression of Cyclin B/Cdc13 results in dissociation of Ccp1 from centromeres. Cells carrying pREP1-empty vector (pREP1-EV) or pREP1-cdc13 were incubated on the minimal pombe minimal glutamate (PMG) medium without thiamine at 30 °C for 22 h. Sad1-mCherry was used as an SPB marker. (Scale bar, 2 μm.) *Right*: Quantification of the percentage of indicated cells with diffuse GFP signal in the nucleus. Experiments were performed in triplicate. At least 40 cells were scored in one single experiment. Error bars represent mean and SD. *P < 0.05. (*F*) Model: the CIM domain of CENP-T is phosphorylated at the onset of mitosis by CDK1, which dissociates Ccp1 from CENP-T, allowing proper positioning of Ndc80C at the adjacent Ndc80 receptor motif. At the end of mitosis, the CIM domain is dephosphorylated by an unknown phosphatase. This leads to the reassociation of Ccp1 with the CIM domain.

Given that the CIM domain and the Ndc80 receptor motif in CENP-T^Cnp20^ are adjacent to each other, we reasoned that the association of Ccp1 with Cnp20-14A during mitosis might interfere with the recruitment of Ndc80C to the Ndc80 receptor motif. To test whether this is the case, we examined closely the effect of *cnp20*-*14A* on the positioning of Ndc80C using cells carrying the GFP-tagged Spc25 and the mCherry-tagged tubulin. In wild type, Ndc80C oscillates along the microtubules between the two SPBs before moving to the two poles (Fig. 7*A*). However, we found that Spc25-GFP appeared not to attach to microtubules in ∼20% of *cnp20*-*14A* mitotic cells, indicating that dephosphorylation of the CIM domain leads to mislocalization of Ndc80C during mitosis (Fig. 7*A*). Importantly, our co-IP results indicated that the interaction between Cnp20-14A and Ndc80 is significantly reduced during mitosis (Fig. 7*B*). Furthermore, since Ccp1 is not present at centromeres in mitosis, we performed co-IP experiments using either cells synchronized in mitosis or unsynchronized cells, which is predominantly at G2. These co-IP experiments showed that the interaction between Ndc80 and CENP-T^Cnp20^ is drastically increased during mitosis (Fig. 7*C*), supporting the idea that the presence of Ccp1 interferes with the interaction between Ndc80 and CENP-T^Cnp20^.

These data suggest that dissociation of Ccp1 from centromeres by phosphorylation of CIM at the onset of mitosis promotes the interaction between CENP-T and Ndc80C, leading to the proper assembly of the outer kinetochores. In fact, we found that *cnp20-14D* has no obvious chromosome missegregation defects (*SI Appendix*, Fig. S17); actually unlike the *cnp20-*Δ*1–55* mutant, *cnp20-14D* is even more resistant to TBZ than WT (Fig. 6*D*). It is likely that the phosphomimetic *cnp20-14D* mutant, but not *cnp20-*Δ*1–55*, can stabilize the interaction between CENP-T and Ndc80. Consistent with this, our co-IP assay showed that Ndc80 interacts more strongly with Cnp20-14D than CENP-T^Cnp20^ (*SI Appendix*, Fig. S18).

### The CIM Domain Is Phosphorylated by CDK1.

We suspected that CDK1 may be responsible for the phosphorylation of the CIM domain in CENP-T^Cnp20^ at the onset of mitosis. To test this, we performed the in vitro kinase assays using human recombinant CDK1-cyclin B and a synthesized peptide derived from Cnp20^1-55^. The highly conserved human CDK1 is known to be capable of functionally replacing the CDK1/Cdc2 in fission yeast (45). A peptide derived from the phospho-null version of the CIM domain, Cnp20^1-55-14A^, was used as a control. We then utilized the mobility shift of phosphorylated proteins on Phos-tag polyacrylamide gel electrophoresis (PAGE) to detect changes in phosphorylation status. We observed migrating bands slower than their nonphosphorylated counterpart from the in vitro assay using Cnp20^1-55^. However, no slow migrating bands were identified in the assay with the Cnp20^1-55-14A^ peptide (Fig. 7*D*). These data demonstrate that CDK1 is capable of phosphorylating the CIM domain of CENP-T^Cnp20^. Various slow migrating bands were observed in the assay with Cnp20^1-55^ (Fig. 7*D*), indicating that the domain contains multiple phosphorylation sites, consistent with our point mutation analysis.

CDK1/Cdc2 in fission yeast is essential for viability. The temperature-sensitive alleles of *cdc2* are arrested at either G2/M or G1/S phase at the restrictive temperature (46), preventing us from assessing its role in Ccp1 regulation in vivo. The mitotic cyclin B is expressed specifically in the G2/M phase and forms the complex with CDK1 to trigger mitosis. We reasoned that if we constitutively overexpressed cyclin B/Cdc13 in fission yeast, CDK1 might phosphorylate the CIM domain of CENP-T^Cnp20^ throughout different stages of the cell cycle, leading to dissociation of Ccp1 from centromeres. Indeed, we found that when Cdc13 is overexpressed in cells carrying GFP-Ccp1, most of the cells displayed high fluorescence signals in the nucleoplasm with weaker GFP-Ccp1 at centromeres, more than 25% in which GFP signals were totally diffuse through the nucleus (Fig. 7*E* and *SI Appendix*, Fig. S19). These data support the idea that the CIM domain is phosphorylated by CDK1. Furthermore, consistent with the functional studies described above, our in vitro binding assay showed that phosphorylation of the CIM domain by CDK1 results in reduced interaction between the CIM domain and Ccp1 (*SI Appendix*, Fig. S20).

## Discussion

Assembly of the outer kinetochore during mitosis is tightly regulated. But the molecular mechanism for this regulation, especially in regional centromeres, remains poorly understood. CENP-T, a key inner kinetochore component, is known to promote the recruitment of Ndc80C, a complex essential for kinetochore–microtubule interactions during mitosis (5, 15–20). Here we found that CENP-T interacts with Ccp1, a conserved homodimer of the NAP family proteins in fission yeast, and demonstrated that the dimeric structure of Ccp1 is required for the interaction. We reported previously that Ccp1 associates with CENP-A and acts as a counteracting CENP-A loading factor to balance the CENP-A and histone H3 at centromeres (36). Interestingly, Ccp1 associates with centromeres at interphase but dissociates from centromeres during mitosis (36, 37). We showed that Ccp1 centromere localization depends on Mis16 (36). In this study, we found that the CENP-T is also required for the Ccp1 centromere localization. In addition, we found that the association of CENP-T^Cnp20^ with centromere depends on Mis16 (*SI Appendix*, Fig. S7). It is possible that Mis16 recruits Ccp1 to centromeres through CENP-T^Cnp20^.

We further identified the first 55 amino acids at the N terminus of CENP-T as the CIM. Notably, the CIM domain is situated next to the Ndc80 receptor motif. We found that the CIM domain can be phosphorylated. Phosphorylation of the CIM domain disrupts the interaction between CENP-T and Ccp1, leading to dissociation of Ccp1 from centromeres. On the other hand, dephosphorylation of the domain results in tight interaction of CENP-T with Ccp1 and consequently the association of Ccp1 with centromeres during mitosis. We further showed that dephosphorylation of CIM disrupts the interaction between CENP-T and Ndc80C. As a result, the phospho-null mutant of CIM exhibits mislocalization of the Ndc80 complex during mitosis and severe chromosome segregation defects. Our data suggest that occupancy of the Ccp1 homodimer at the CIM domain during interphase prevents the interaction of the adjacent Ndc80 receptor motif of CENP-T with Ndc80C. Removing Ccp1 from CENP-T makes the Ndc80 receptor motif accessible during mitosis, which may allow Ndc80C to interact with CENP-T to be properly assembled. Nevertheless, we found that only ∼20% of the *cnp20-14A* cells displayed mislocalized Scp25-GFP signal and the mutant cells are viable, suggesting that alternative mechanisms are also used in recruiting the Ndc80 complex during mitosis.

We found that multiple amino acids in the CIM domains are conserved across different species, especially in *S. pombe*, *M. musculus*, and *H. sapiens* (*SI Appendix*, Fig. S11). The Ndc80 receptor motif which is next to the CIM domain is considered as a conserved domain for the interaction between the Ndc80 complex and CENP-T by previous studies (15, 16, 21–24). Phosphorylation of the Ndc80 receptor motif is required for the interaction (16, 21–24). Based on our sequence alignment analysis, there are also a few key residues conserved in the motif (*SI Appendix*, Fig. S11). It is likely that both the Ndc80 receptor motif and the Ndc80 complex coevolve during evolution to maintain the interaction. Similar to the Ndc80 receptor motif, we believe that the CIM domain is also functionally conserved.

We used both the yeast two-hybrid system and in vitro pull-down assays to confirm the interaction between the CIM domain and Ccp1. Nevertheless, we noted that our yeast two-hybrid results are not completely consistent with our in vitro pull-down data. We think it is likely due to the fact that two assays were performed in two very different systems. Unlike the yeast two-hybrid system, the pull-down assays were performed in the nonstringent conditions with much higher protein levels. Also, the truncated BD-Cnp20-N in our yeast two-hybrid assays showed interactions with both AD (empty vector) and AD-Ccp1-4A (the homodimer mutant), but the full-length BD-Cnp20 did not show any interactions with AD or AD-Ccp1-4A (*SI Appendix*, Fig. S9). We believe that the truncated BD-Cnp20-N is likely self-activating. On the other hand, the self-activation activity of full-length BD-Cnp20 may be inhibited by the configuration of the full-length CENP-T^Cnp20^ protein.

Our in vitro kinase assays further demonstrated that the CIM domain is phosphorylated by CDK1. This was further supported by the fact that constitutive overexpression of Cdc13/Cyclin B results in dissociation of Ccp1 from centromeres. Presumably, overexpression of Cdc13 causes CENP-T to be phosphorylated during interphase, which disrupts the interaction between the CIM domain of CENP-T and Ccp1, a phenomenon that is similarly observed in the Cnp20-14D mutant. Our in vitro kinase assays indicated that the domain contains multiple phosphorylation sites. Consistent with this, our point mutation analysis revealed that phosphorylation of all of the 14 potential phosphorylation sites within CIM is required for the dissociation of Ccp1 from centromeres during mitosis. It has been shown that CDK1 in multiple species specifically phosphorylates the N terminus of CENP-T, especially the Ndc80 receptor motif, during mitosis (16, 21–24). Considering the conserved role of CENP-T and the close distance between the CIM domain and the Ndc80 receptor motif, it is likely that the CIM domain is also phosphorylated by CDK1 in the same cell cycle–regulated manner. Nevertheless, we found that only four of 14 phosphorylation sites in the CIM domain of CENP-T are located within known CDK1 consensus motifs. Overexpression of Cdc13 also only partially dissociates Ccp1 from centromeres. These data suggest that additional kinases are responsible for phosphorylation of the CIM domain. Consistent with this idea, our in vitro pull-down assay showed that phosphorylation of the CIM domain by CDK1 results in only partial loss of its interaction with Ccp1.

Our study uncovers a previously unrecognized mechanism behind the regulation of kinetochore organization in regional centromeres. This work also provides a molecular explanation for the observed Ccp1 dissociation from centromeres during mitosis. Based on our data, we propose the following model: The CIM domain of CENP-T is phosphorylated at the onset of mitosis by CDK1. The phosphorylation expels Ccp1 from CENP-T, which allows the Ndc80 receptor motif to interact with Ndc80C. At the end of mitosis, the CIM domain is dephosphorylated by an unknown phosphatase. This leads to the reassociation of Ccp1 with the CIM domain (Fig. 7*F*), which blocks the binding of Ndc80C to CENP-T. Similar to Ccp1, some other centromeric proteins, including HJURP/Scm3, Mis16, Mis18, Eic1/Mis19/Kis1, and Eic2/Mis20 (41, 47–52) also dissociate from centromeres during mitosis. The mechanism of competitive exclusion between two proteins through phosphorylation identified here may be a general principle also used by these centromeric proteins to promote kinetochore assembly.

Whereas the point centromere in *S. cerevisiae* contains a single nucleosome (53, 54), CENP-A–containing nucleosomes at regional centromeres are interspersed with canonical histone H3–containing nucleosomes. The histone H3 at centromeres is important for centromeric localization of CENP-A (55, 56). The CENP-T complex has been shown to be associated with histone H3 and DNA in centromeres (5, 25, 27). CENP-T/W/S/X can form nucleosome-like structure in vitro (25, 26). How the CENP-T particles and CENP-A nucleosome in centromeres are coordinated to establish homeostasis is unknown. Ccp1 counteracts the loading of CENP-A to ensure proper balance of CENP-A and H3 in centromeres as well as removing mislocalized CENP-A at ectopic sites (36). In this study, we found that CENP-T interacts with Ccp1 and is required for Ccp1 localization. Deletion of the CIM domain of CENP-T results in dissociation of Ccp1 from centromeres. Similar to the *ccp1Δ* mutant, the CIM-deleted mutant is sensitive to TBZ and also exhibited increased silencing in centromeres, indicative of an increased amount of CENP-A nucleosomes in the region. We propose that CENP-T recruits Ccp1 to centromeres at the end of mitosis to remove excessive CENP-A nucleosomes and allow room for the incorporation of sufficient CENP-T–containing particles to centromeres.

## Materials and Methods

Standard media and genetic analysis for fission yeast were used (57). The temperature-sensitive allele of *cnp20* was created by marker reconstitution mutagenesis (58). Details of the materials and methods used in this study, including mutant generation, immunoprecipitation, TAP-tag purification, yeast two-hybrid assays, in vitro pull-down assays, microscopy, in vitro kinase assays, and phos-tag PAGE are provided in *SI Appendix*, *SI Materials and Methods*. Fission yeast strains used in this study are listed in SI Appendix, Table S1.

## Supplementary Material

Supplementary File

Supplementary File

## Data Availability

All study data are included in the article and/or supporting information.
