# Supplementary material for: Ccp1-Ndc80 switch at the N terminus of CENP-T regulates kinetochore assembly
Source: Proc Natl Acad Sci U S A. 2021 Nov 22;118(48):e2104459118. doi: 10.1073/pnas.2104459118 (PMC8640933; doi:10.1073/pnas.2104459118)
Supplement: Supplementary File [file pnas.2104459118.sapp.pdf]

Supplemental Information

Ccp1-Ndc80 switch at the N-terminus of CENP-T regulates kinetochore assembly

Qianhua Dong, Xue-lei Liu, Xiao-hui Wang, Yu Zhao, Yu-hang Chen, Fei Li

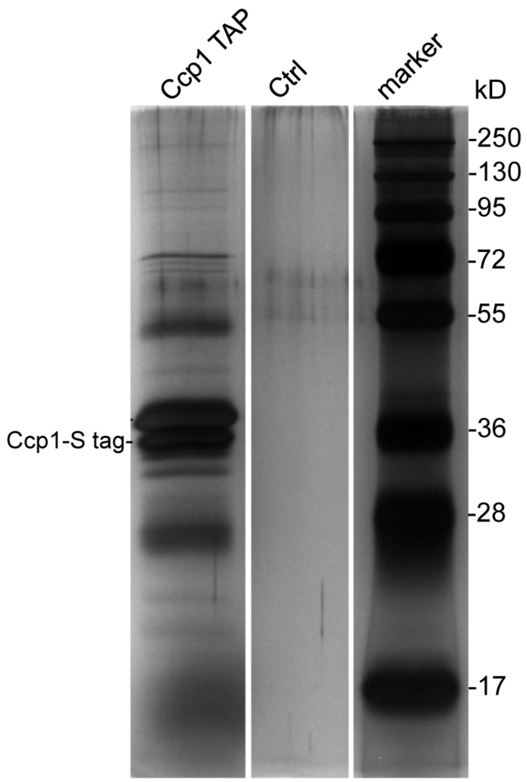

Figure S1. Silver-stained SDS-PAGE gel showing the TAP-tag purification of Ccp1 and a control purification. Molecular weights are indicated in kD. Ctrl, control.

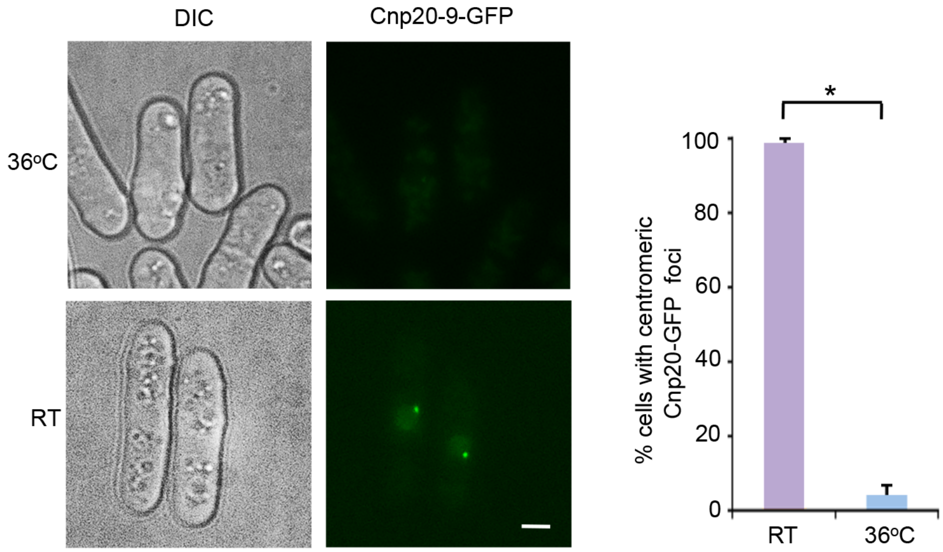

Figure S2. Cnp20-9-GFP dissociates from centromeres in *cnp20-9* mutant at the restrictive temperature. The native *cnp20*$^{+}$ was replaced by *cnp20-9-GFP* with its endogenous promoter. RT, room temperature. Scale bar, 2 μm. Right: quantification of the percentage of cells with centromeric GFP signal. Experiments were performed in triplicate. At least 40 cells were scored in one single experiment. Error bars represent mean and SD. *, $p<0.05$.

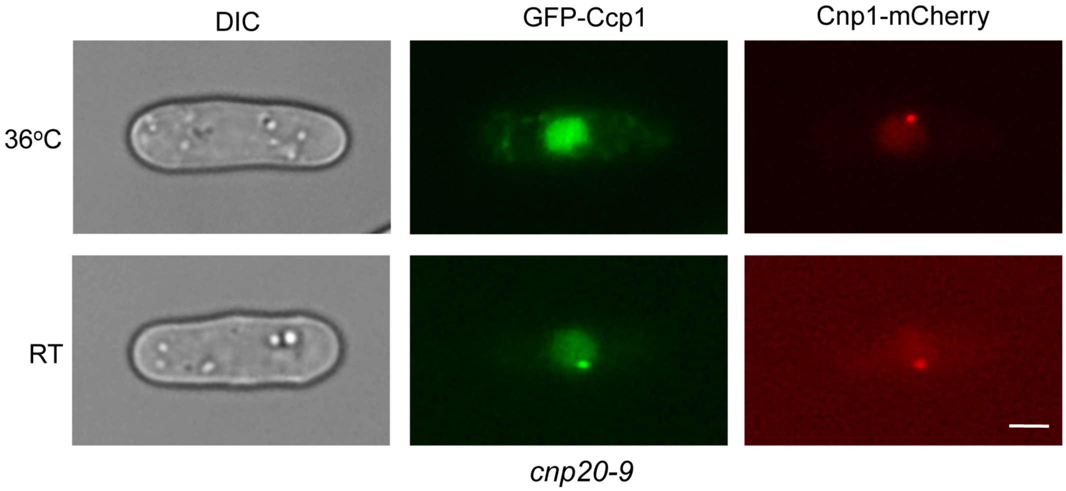

Figure S3. The centromeric association of Ccp1 is lost in *cnp20-9* at the restrictive temperature. The *cnp20-9* cells were incubated at 36°C for 6 hrs. CENP-A^{Cnp1}-mCherry was used as a centromere marker. RT, room temperature. Scale bar, 2 μm.

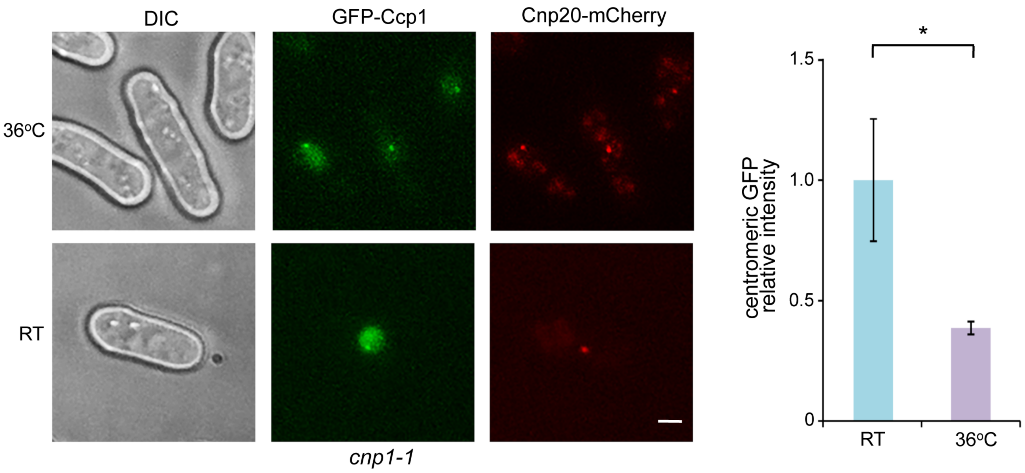

Figure S4. The centromere localization of GFP-Ccp1 is partially lost in the *cnp1-1* mutant at the restrictive temperature. CENP-T^{Cnp20}-mCherry was used as a centromere marker. RT, room temperature. Right: Quantification of relative fluorescence intensity of centromeric GFP-Ccp1. At least 50 cells were scored in one single experiment. Error bars represent mean and SD. *, $p<0.05$. Scale bars, 2 μm.

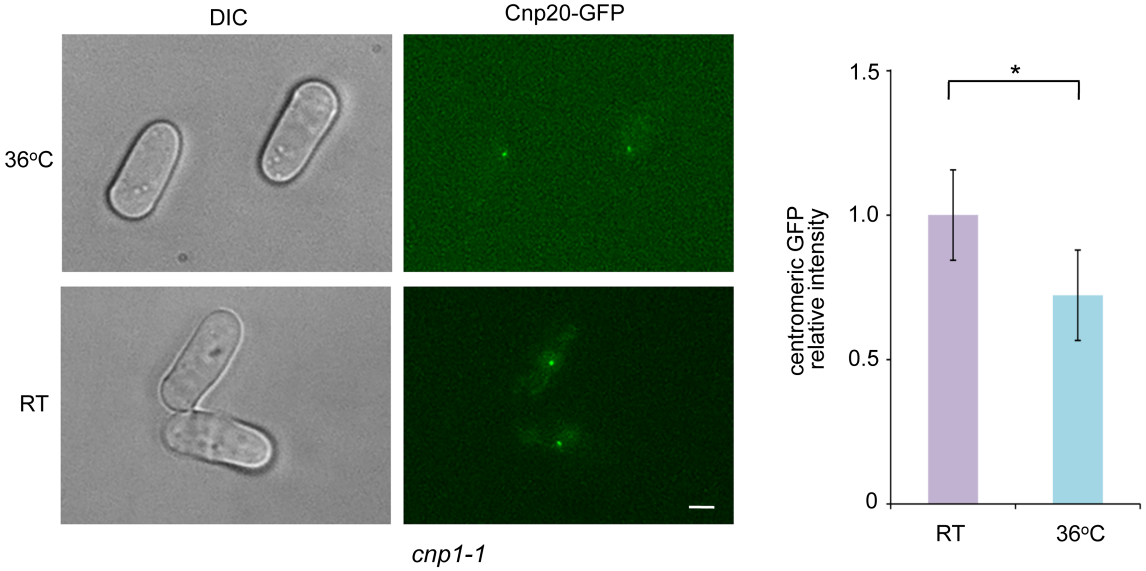

Figure S5. The association of CENP-T^{Cnp20}-GFP with centromeres is reduced in the *cnp1-1* mutant at the restrictive temperature. RT, room temperature. Right: Quantification of relative fluorescence intensity of centromeric CENP-T^{Cnp20}-GFP. At least 40 cells were scored in one single experiment. Error bars represent mean and SD. *, $p<0.05$. Scale bars, 2 μm.

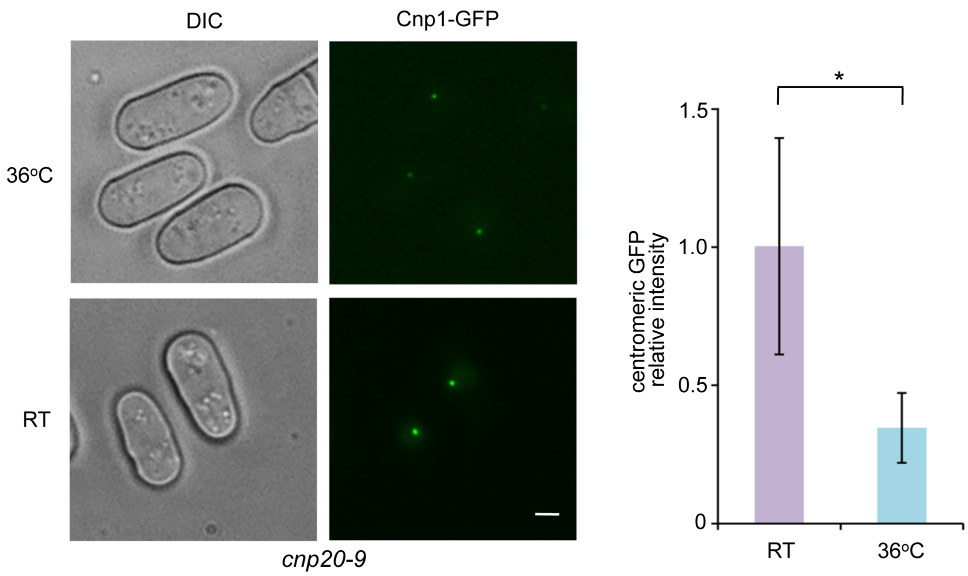

Figure S6. The association of CENP-A^{Cnp1}-GFP with centromeres is reduced in the *cnp20-9* mutant at the restrictive temperature. RT, room temperature. Right: Quantification of relative fluorescence intensity of centromeric CENP-A^{Cnp1}-GFP. At least 40 cells were scored in one single experiment. Error bars represent mean and SD. *, $p<0.05$. Scale bars, 2 μm.

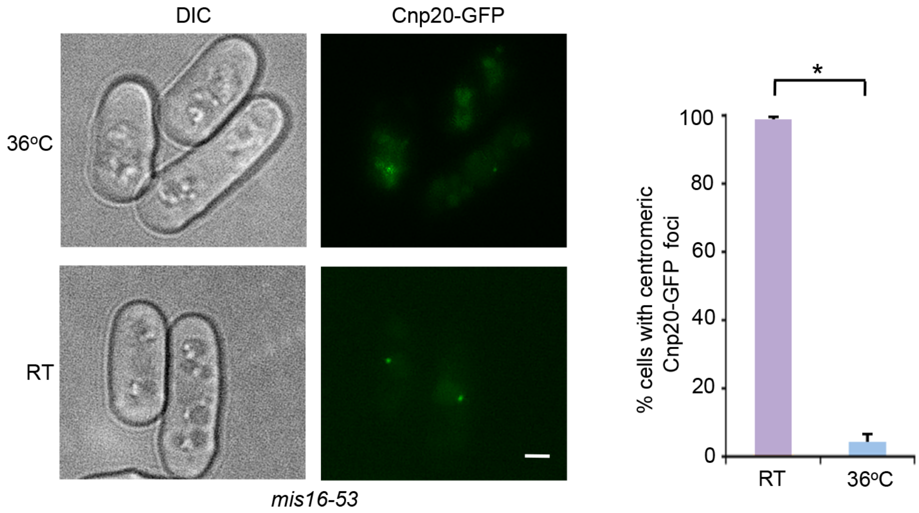

Figure S7. The association of CENP-T^{Cnp20}-GFP with centromeres is disrupted in the *mis16-53* mutant at the restrictive temperature. RT, room temperature. Scale bar, 2 μm. Right: quantification of the percentage of cells with centromeric CENP-T^{Cnp20}-GFP signal. Experiments were performed in triplicate. At least 40 cells were scored in one single experiment. Error bars represent mean and SD. *, $p<0.05$.

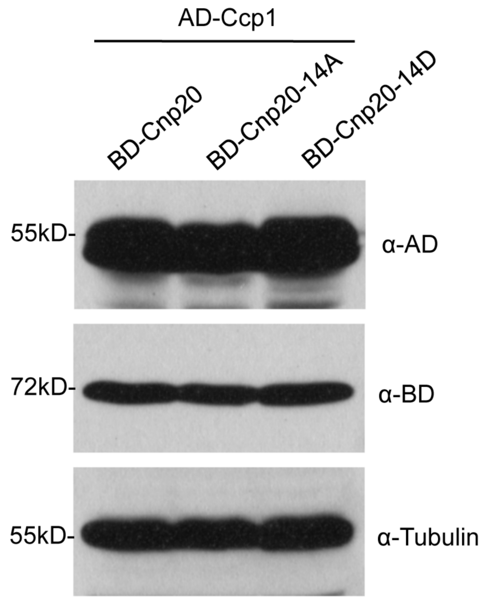

Figure S8. Western blot analysis of cell extracts from *Saccharomyces cerevisiae* cells expressing AD-Ccp1 with BD- CENP-T^{Cnp20}, BD-Cnp20-14A, or BD- Cnp20-14D. Tubulin was used as a control.

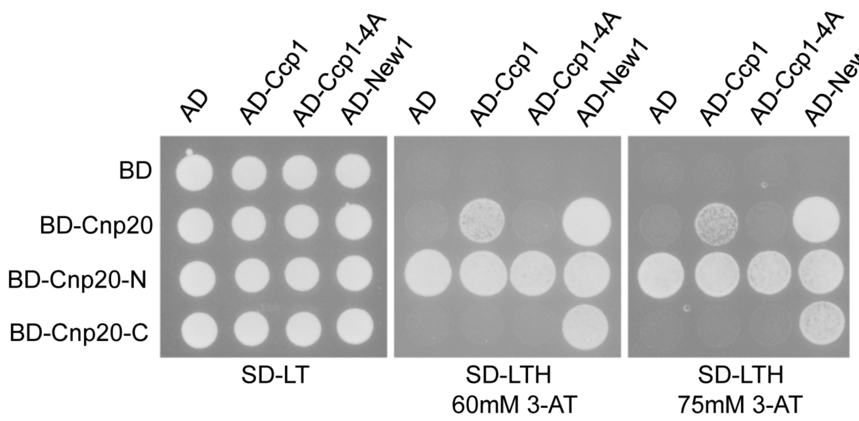

Figure S9. The Y2H analysis showed that Ccp1 interacts with the N-terminus of CENP-T^{Cnp20}. Cnp20-N: aa 1-368. Cnp20-C: aa 369-479. *Saccharomyces cerevisiae* strains carrying the indicated plasmid combinations were spotted onto SD minimal medium plates lacking either Leu and Trp (SD-LT) or Leu,Trp and His (SD-LTH) supplemented with either 60mM 3-amino-1,2,4-triazole (3-AT) or 75mM 3-AT, and grew at 30°C.

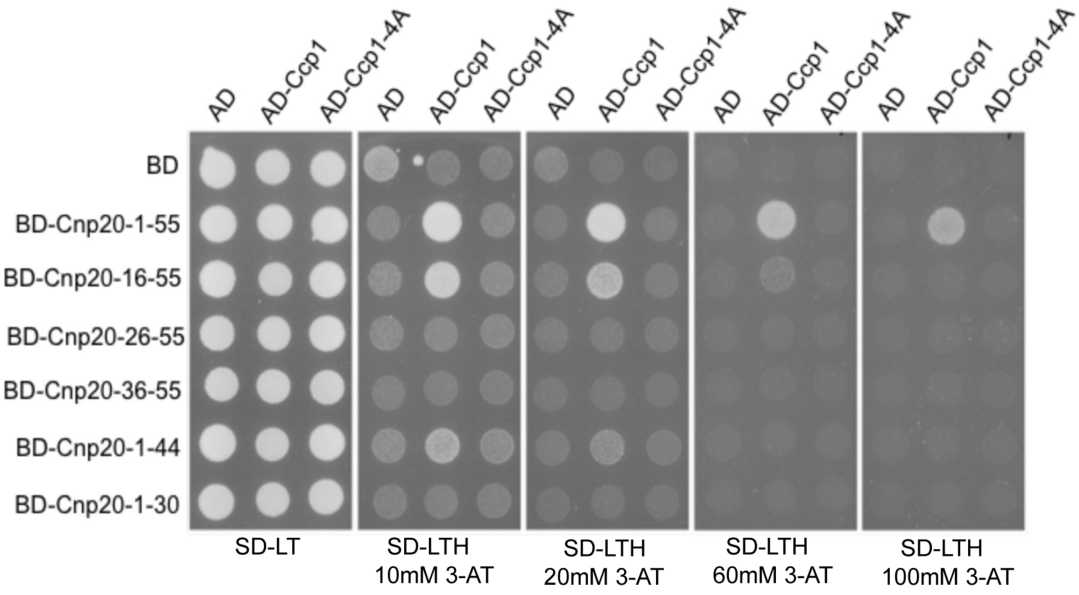

Figure S10. The Y2H analysis showed that the first 55 aa of CENP-T^{Cnp20} was the minimum Ccp1-interacting motif. *Saccharomyces cerevisiae* strains carrying the indicated plasmid combinations were spotted onto SD minimal medium plates lacking either Leu and Trp (SD-LT) or Leu,Trp and His (SD-LTH) supplemented with indicated concentrations of 3-AT, and grew at 30°C.

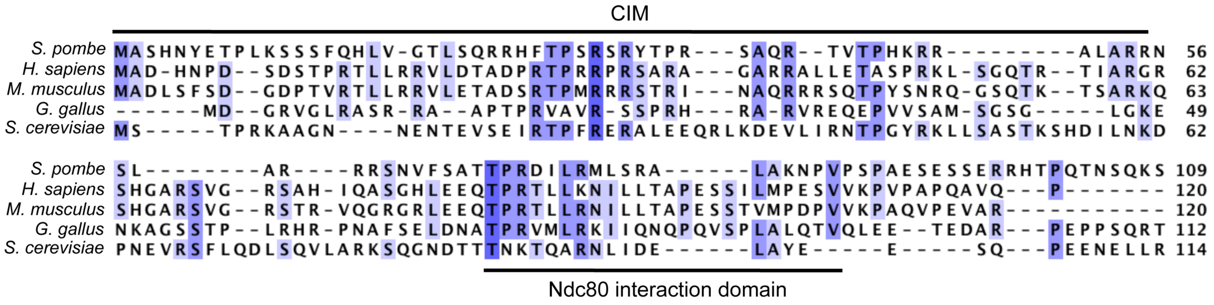

Figure S11. Sequence alignment of the CIM domain of *S. pombe* CENP-T^{Cnp20} (UniProt ID: Q9HGK9) with CENP-T orthologs in *Gallus gallus* (UniProt ID: F1NPG5), *Homo sapiens* (UniProt ID: Q96BT3), and *Mus musculus* (UniProt ID: Q3TJM4), and *S. cerevisiae* (UniProt ID: P43618).

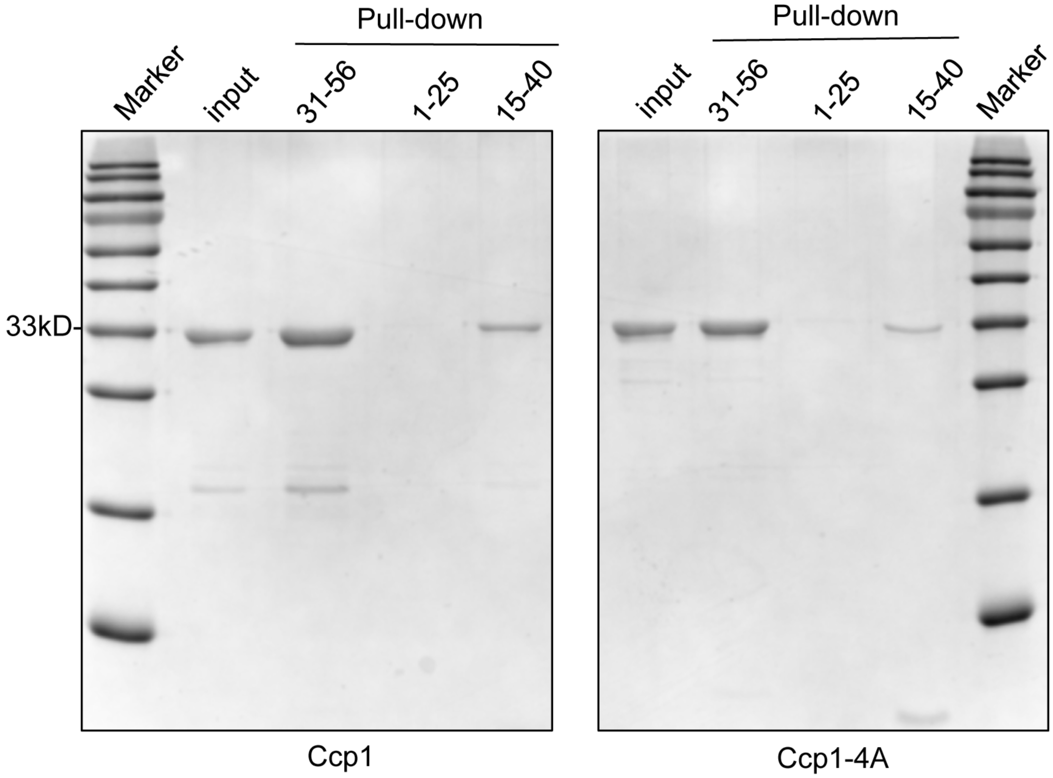

Figure S12. *in vitro* pull-down assays demonstrate that Ccp1 interacts with peptides derived from Cnp20$^{15\text{-}40}$ and Cnp20$^{31\text{-}56}$, but the interaction of these peptides with Ccp1-4A is reduced. Purified full-length Ccp1/Ccp1-4A and three peptides derived from Cnp20$^{1\text{-}25}$, Cnp20$^{15\text{-}40}$ and Cnp20$^{31\text{-}56}$ were used.

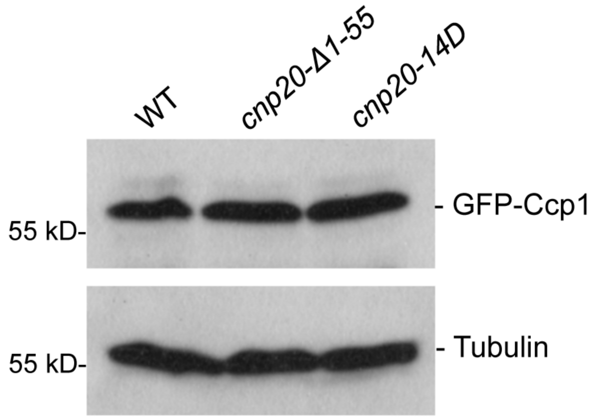

Figure S13. Western blot analysis of cell extracts from indicated cells expressing GFP-Ccp1. Tubulin was used as a control.

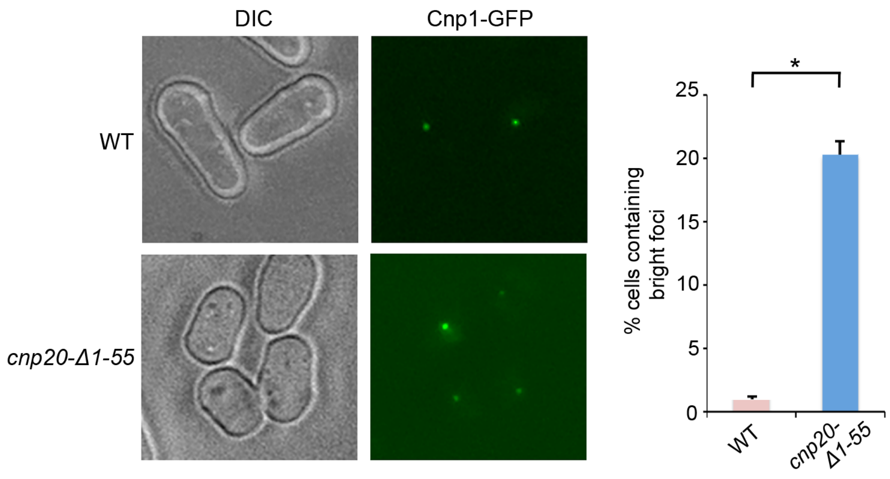

Figure S14. CENP-A^{Cnp1}-GFP signal in centromeres is increased in a subpopulation of c*np20*-Δ*1-55*. WT, wild type. Scale bar, 2 μm. Right: the percentage of cells exhibiting a single GFP signal with an intensity twice the average of wild-type was plotted. Experiments were performed in triplicate. At least 40 cells were scored in one single experiment. Error bars represent mean and SD. *, $p<0.05$.

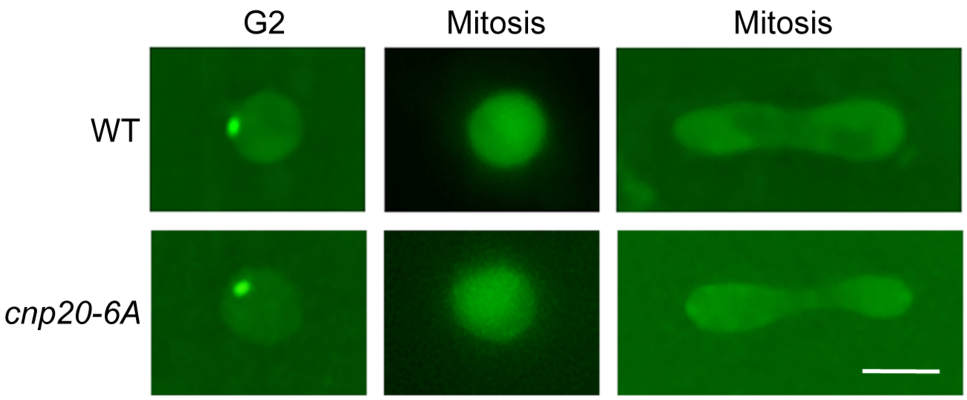

Figure S15. The distribution pattern of GFP-Ccp1 during mitosis in the *cnp20-6A* mutant. Scale bar, 2 μm.

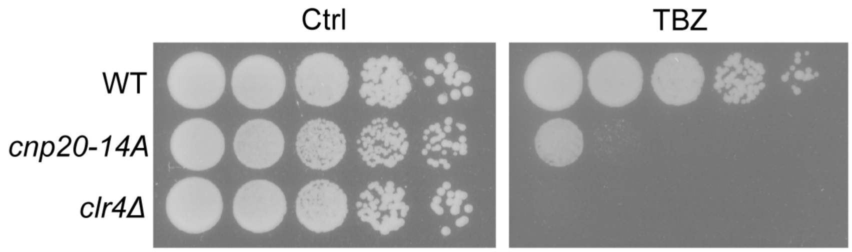

Figure S16. The *cnp20-14A* mutant is sensitive to TBZ. Indicated cells grew on the rich YES medium in the absence or presence (15 μg/ml) of TBZ at 30°C for 3 to 5 days.

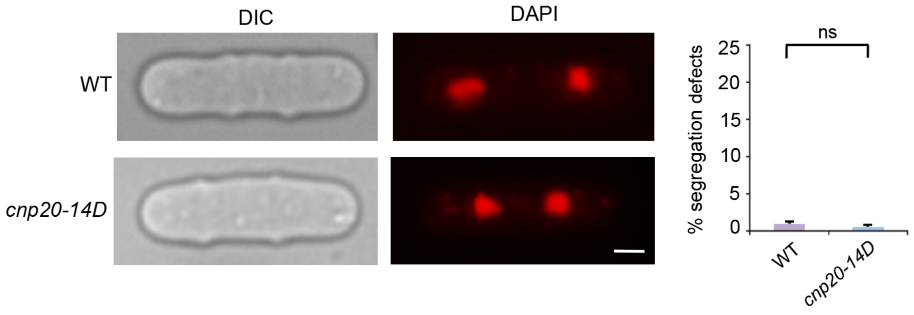

Figure S17. The *cnp20-14D* mutant cells display no obvious chromosome mis-segregation defects during mitosis, as shown by DAPI staining. Scale bar, 2 μm. Right, the percentage of mitotic cells showing lagging chromosomes. *n*, number of cells counted. Experiments were performed in triplicate. At least 40 cells were scored in one single experiment. Error bars represent mean and SD. *; ns, no significant differences

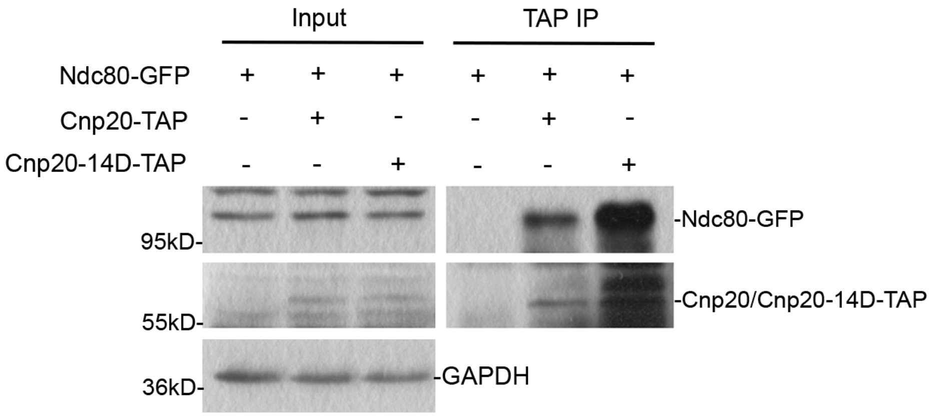

Figure S18. Lysates from indicated cells synchronized at metaphase using *nda3*-KM311 were immunoprecipitated with an antibody specific for TAP. Precipitated proteins were analyzed by Western blotting using indicated antibodies. GAPDH was used a loading control.

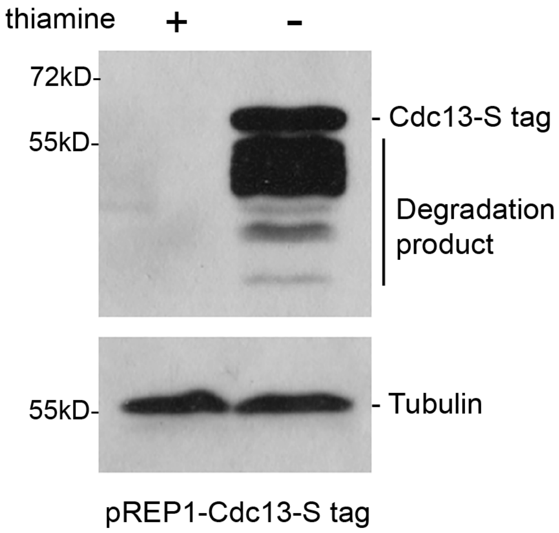

Figure S19. Western blot analysis of cell extracts from cells overexpressing Cdc13-S tag. Cells carrying pREP1-Cdc13- S tag were incubated on the minimal PMG medium with (control) or without thiamine (overexpression) at 30°C for 22 hrs. Tubulin was used as a control.

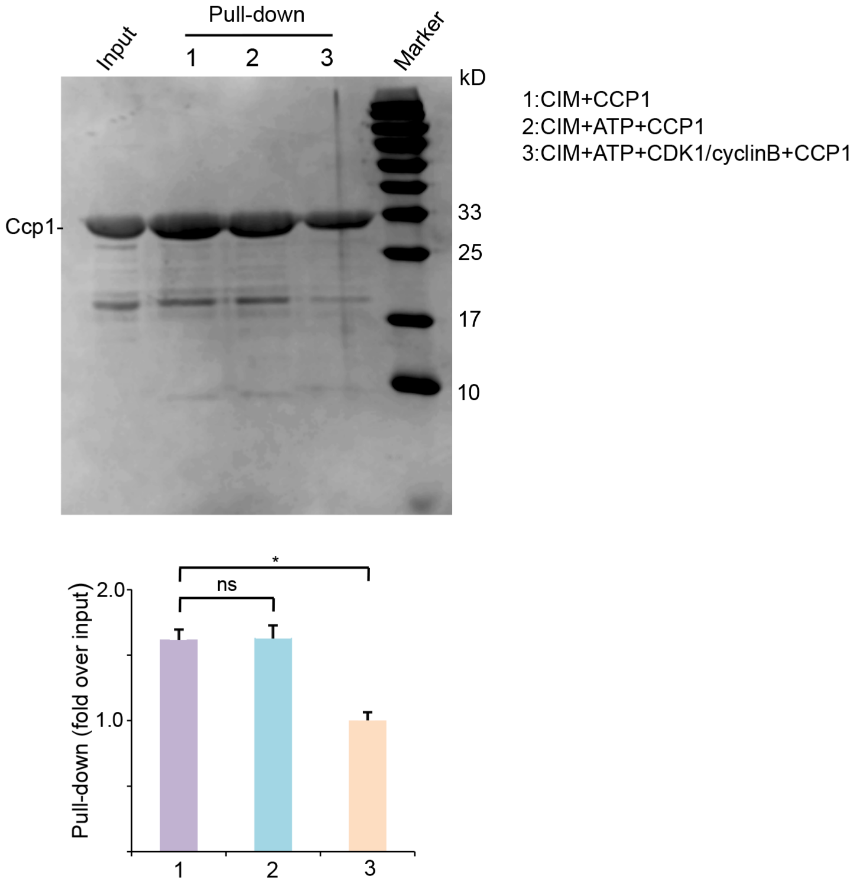

Figure S20. *in vitro* pull-down assays demonstrates that phosphorylation of the CIM domain of CENP-T^{Cnp20} weakened the interaction of the domain with Ccp1. Purified full-length Ccp1 and a peptide derived from $Cnp20^{1\text{-}55}$ were used. Bottom panel: quantification of the pull-down efficiency. The signals from western blotting were normalized to inputs. The band intensity was quantified with ImageJ. Two independent experiments were performed for the quantification. Error bars represent mean and SD. *, $p<0.05$. ns, no significant differences.

Supporting Materials and Methods

Mutant generation

The temperature-sensitive allele of *cnp20* was created by marker reconstitution mutagenesis (1). The PCR-mediated random mutation of *cnp20*$^+$ was performed by using GeneMorph II random mutagenesis kit (200550, Agilent Technologies, Inc.). The randomly mutagenized *cnp20*$^+$ was transformed to wild-type cells to replace the native *cnp20*$^+$. The candidate temperature-sensitive colonies were identified by incubating the replicated colonies on YES with 5 mg/L phloxine B (P4030, Sigma) at 36°C overnight. The temperature sensitivity of the mutants was further confirmed by growth assays at 36°C. Mutations within the temperature-sensitive *cnp20* alleles were identified by sequencing. The mutants identified were backcrossed to wild type three times to obtain a clean background. The temperature sensitive allele *cnp20-9* can be complemented by an ectopic copy of *cnp20*$^+$ in the *ade6*$^+$ locus.

CRISPR/Cas9-mediated gene editing was performed as described (2). The synthesized DNA oligos containing the gRNA sequence were inserted into the plasmid pYZ033 by NEBuilder HiFi DNA assembly kit (E2621S, NEB). The donor DNA was prepared by overlapping PCR. The Cas9 plasmid and the donor DNA were co-transformed into the yeast strains by electroporation. Colony PCRs followed by restriction enzyme digestion were performed to identify the candidate colonies containing the mutation. The candidate colonies grew in YES overnight to get rid of the Cas9 plasmid.

Immunoprecipitation

Immunoprecipitation was performed as previously described (3) with minor modifications. Cells were grown in YES rich media at 30 ^{o}C until the mid-log phase. For mitotic arrest, strains carrying the cold-sensitive *nda3*-KM311 were used. Cells carrying *nda3*-KM311 were incubated at 30 ^{o}C overnight to mid-log phase, and then shifted to 18 ^{o}C for another 8 hrs to arrest in mitosis (4). Cells were collected and lysed in lysis buffer (50 mM Tris-HCl at pH 7.5, 1 mM EDTA, 150 mM NaCl, 0.5% Nonidet P-40 and 10% Glycerol) with 1 mM PMSF, proteinase inhibitor (P8215; Sigma), 1 mM Na_3VO_4, and 1mM β-glycerolphosphate. The cell lysis was further sonicated with a tip sonicator three times. Cell extracts were then incubated with IgG sepharose (17-0969-01, GE Healthcare) at 4 °C for 1 h. After washing with lysis buffer three times, 5 minutes each time on the rotate at 4 °C, proteins were eluted in SDS loading buffer. Eluates were analyzed by Western blotting using commercial anti-TAP HRP (MA1-108-HRP, Thermo Fisher Scientific), anti-GFP HRP (ab190584, Abcam), anti-Tubulin (ab6160, Abcam), anti-GAPDH (ab9485, Abcam) antibodies.

TAP-tag purification

TAP-tag purification was conducted as described previously (5). Briefly, lysates from cells carrying Ccp1-TAP were cleared by centrifugation and incubated with IgG Sepharose 6 Fastflow beads (CL6B200, Amersham Bioscience) for 2 hr. After washing, protein was eluted from the beads and incubated overnight with TEV protease (12575-015, Invitrogen). Supernatant was then further purified by S-protein agarose (69704, Novagen) and incubated for 3 hr at 4°C. Complexes were eluted with 8 M urea, and subjected to mass spectrometry at Proteomics Resource Center at NYU School of Medicine (New York, NY).

Yeast two-hybrid assays

Yeast two-hybrid analyses were performed as described (6). Briefly, bait and prey plasmids were co-transformed into *Saccharomyces cerevisiae* strain MaV203 (PQ10001-01, Invitrogen). Positive interactions were determined by growth on synthetic defined medium lacking leucine, tryptophan, histidine (SD-LTH) and containing different concentrations of 3-amino-1,2,4-triazole (3-AT) (A8056, Sigma).

***in vitro* pull down assays**

The *in vitro* pull-down assays were performed as previously described (7) with minor modification. 20 µl of streptavidin agarose beads were incubated with biotinylated peptides (1mg/mL) for 1 hr at 4°C. After washing with washing buffer (20 mM Hepes pH 7.0, 200 mM NaCl, 1% Glycerol), the beads were incubated with purified Ccp1 (1.2 mg/mL) for 30 min at 4°C. The beads were then washed three times with buffer containing 20 mM Hepes, pH 7.0, 200 mM NaCl, 1% Glycerol. The pull-down protein was subsequently analyzed by SDS-PAGE and coomassie blue staining.

Microscopy

Microscopy was performed as described (8). Cells were imaged using the Delta Vision System (Applied Precision, Issaquah, WA). Images were taken as z-stacks of 0.2 µm increments with an oil immersion objective (x100), and deconvolved using SoftWoRX2.50 software (Applied Precision). Quantification of the signals was conducted by using ImageJ (v1.53a, NIH) as described previously (9). To quantify GFP signals in cells, a single in-focus plane was acquired. Using ImageJ, an outline was drawn around each GFP spot and circularity, area, mean

fluorescence measured, along with several adjacent background readings. The total corrected cellular fluorescence (TCCF) = integrated density – (area of selected cell × mean fluorescence of background readings), was calculated. This TCCF was then equalized against the mean TCCF of wild type cells, with results presented as a fold increase over wild type levels.

***in vitro* kinase assays**

in vitro kinase assays were performed according to the manufacturer's instructions (14-450, Millipore). Briefly, synthesized peptides were incubated with CDK1/cyclinB (14-450, Millipore) in 5 µl 5x reaction buffer (40mM MOPS/NaOH pH7.0, 1 mM EDTA) at 30 ^{o}C for 10 min. The reaction was terminated by boiling and analyzed by Phos-tag PAGE.

Phos-tag PAGE

Phos-tag PAGE was performed according to Phos-tag^{TM} SDS-PAGE Guidebook. 20 µl of samples were loaded to 18% Phos-tag PAGE (100 µM Phos-tag acrylamide (304-93521, Wako Chemicals USA, Inc.), 200 µM $MnCl_2$) without SDS. The gel was run under a constant current (25mA/gel) and analyzed by coomassie brilliant blue.

Supplemental Data Table S1. Strains used in this study

Strain name	Genotype
FL360	h^{-} nda3-KM311
FL486	h^{90} ndc80::ndc80-GFP-HA-kan ade6-M216 leu1-32 lys1-131 ura4-D18
FL543	h^{-} ccp1Δ::$ura4^{+}$ leu1-32 ade6-210 ura4-D18
FL669	h^{-} ccp1::ccp1-GFP-kanMX6 leu1-32 ade6-M210 ura4-D18
FL691	h^{-} ccp1::GFP-ccp1-kanMX6 leu1-32 ade6-M210 ura4-D18
FL694	h^{+} cnp20::cnp20-GFP-kanMX6 leu1-32 ade6-M210 ura4-D18
FL813	h^{90} spc25::spc25-GFP-HA-kan ade6-M216 leu1-32 lys1-131 ura4-D18
FL815	h^{90} Z<<Padh13-mCherry-atb2+<<nat leu1-32 ade6-M210
FL816	h^{-} cnt2::$ade6^{+}$-natMX6 ade6Δ::hphMX6 leu1-32 ura4-D18
FL820	h^{-} cnp20-9-$ura4^{+}$-$his5^{+}$ leu1-32 ura4-D18 his5 D ade6-M210
FL821	h^{+} cnp20-9-$ura4^{+}$-$his5^{+}$ leu1-32 ura4-D18 his5 D ade6-M210
FL827	h^{-} cnp20::cnp20-TAP-kanMX6 leu1-32 ade6-M210 ura4-D18
FL828	h^{-} cnp20::cnp20-TAP-nat leu1-32 ade6-M210 ura4-D18
FL833	$h^{?}$ cnp20::cnp20-TAP-nat ccp1:: ccp1-GFP-kanMX6 leu1-32 ade6-M210 ura4-D18
FL834	h^{-} cnp20-9-$ura4^{+}$-$his5^{+}$ $ade6^{+}$::$cnp20^{+}$ leu1-32 ura4-D18 his5 D
FL835	$h^{?}$ cnp20-9-$ura4^{+}$-$his5^{+}$ ccp1::GFP-ccp1-kanMX6 leu1-32 ade6-M210 ura4-D18
FL836	$h^{?}$ ccp1Δ:: $ura4^{+}$ cnp20::cnp20-GFP-kanMX6 leu1-32 ade6-M210 ura4-D18
FL837	$h^{?}$ cnp20-9-$ura4^{+}$-$his5^{+}$ ccp1::GFP-ccp1-kanMX6 $ade6^{+}$::$cnp20^{+}$ leu1-32 ade6-M210 ura4-D18
FL838	$h^{?}$ cnp20-9-$ura4^{+}$-$his5^{+}$ ccp1::GFP-ccp1-kanMX6 $ade6^{+}$::cnp20Δ1-30 leu1-32 ade6-M210 ura4-D18
FL839	$h^{?}$ cnp20-9-$ura4^{+}$-$his5^{+}$ ccp1::GFP-ccp1-kanMX6 $ade6^{+}$::cnp20Δ1-55 leu1-32 ade6-M210 ura4-D18
FL840	$h^{?}$ cnp20-9-$ura4^{+}$-$his5^{+}$ ccp1::GFP-ccp1-kanMX6 $ade6^{+}$::cnp20Δ1-88 leu1-32 ade6-M210 ura4-D18
FL841	h^{-} cnp20Δ1-30 leu1-32 ade6-M210 ura4-D18 his3-D1

FL842	h^- cnp20Δ1-55 leu1-32 ade6-M210 ura4-D18 his3-D1
FL843	$h^?$ cnp20Δ1-30 ccp1::GFP-ccp1-kanMX6 leu1-32 ade6-M210 ura4-D18 his3-D1
FL844	$h^?$ cnp20Δ1-55 ccp1::GFP-ccp1-kanMX6 leu1-32 ade6-M210 ura4-D18 his3-D1
FL845	$h^?$ cnp20Δ2-45 ccp1::GFP-ccp1-kanMX6 leu1-32 ade6-M210 ura4-D18 his3-D1
FL846	$h^?$ cnp20Δ31-55 ccp1::GFP-ccp1-kanMX6 leu1-32 ade6-M210 ura4-D18 his3-D1
FL849	$h^?$ ccp1Δ:: ura4$^+$ cnt2::ade6$^+$-natMX6 ade6Δ::hphMX6 leu1-32 ura4-D18
FL851	$h^?$ cnp20Δ1-55 cnt2::ade6$^+$-natMX6 ade6Δ::hphMX6 leu1-32 ura4-D18
FL853	$h^?$ cnp20-14D cnt2::ade6$^+$-natMX6 ade6Δ::hphMX6 leu1-32 ura4-D18
FL855	$h^?$ cnp20-14D ccp1::GFP-ccp1-kanMX6 leu1-32 ade6-M210 ura4-D18 his3-D1
FL856	$h^?$ cnp20-12D$^{3-14}$ ccp1::GFP-ccp1-kanMX6 leu1-32 ade6-M210 ura4-D18 his3-D1
FL857	$h^?$ cnp20-9D$^{6-14}$ ccp1::GFP-ccp1-kanMX6 leu1-32 ade6-M210 ura4-D18 his3-D1
FL858	$h^?$ cnp20-6D$^{9-14}$ ccp1::GFP-ccp1-kanMX6 leu1-32 ade6-M210 ura4-D18 his3-D1
FL859	$h^?$ cnp20-6D$^{6-11}$ ccp1::GFP-ccp1-kanMX6 leu1-32 ade6-M210 ura4-D18 his3-D1
FL860	$h^?$ cnp20-5D$^{6-10}$ ccp1::GFP-ccp1-kanMX6 leu1-32 ade6-M210 ura4-D18 his3-D1
FL861	$h^?$ cnp20-3D$^{6-8}$ ccp1::GFP-ccp1-kanMX6 leu1-32 ade6-M210 ura4-D18 his3-D1
FL862	$h^?$ cnp20-3D$^{9-11}$ ccp1::GFP-ccp1-kanMX6 leu1-32 ade6-M210 ura4-D18 his3-D1
FL863	$h^?$ cnp20-14A ccp1::GFP-ccp1-kanMX6 leu1-32 ade6-M210 ura4-D18 his3-D1
FL864	$h^?$ cnp20-6A$^{6-11}$ ccp1::GFP-ccp1-kanMX6 leu1-32 ade6-M210 ura4-D18 his3-D1
FL865	h^- cnp20-14D leu1-32 ade6-M210 ura4-D18 his3-D1
FL866	h^- cnp20-6D$^{6-11}$ leu1-32 ade6-M210 ura4-D18 his3-D1
FL867	h^- cnp20-14A leu1-32 ade6-M210 ura4-D18 his3-D1
FL868	h^- cnp20-6A$^{6-11}$ leu1-32 ade6-M210 ura4-D18 his3-D1
FL869	$h^?$ sad1::sad1-mCherry-nat spc25::spc25-GFP-HA-kan leu1-32
FL870	$h^?$ cnp20-14A sad1::sad1-mCherry-nat spc25::spc25-GFP-HA-kan leu1-32
FL871	$h^?$ spc25::spc25-GFP-HA-kan Z<<Padh13-mCherry-atb2$^+$<<nat leu1-32
FL872	$h^?$ cnp20-14A spc25::spc25-GFP-HA-kan Z<<Padh13-mCherry-atb2$^+$<<nat leu1-32

FL873	$h^{?}$ pREP1 *sad1::sad1-mCherry-nat ccp1::GFP-ccp1-ura4*$^{+}$ *leu1-32 ura4-D18*
FL874	$h^{?}$ pREP1-*cdc13 sad1::sad1-mCherry-nat ccp1::GFP-ccp1-ura4*$^{+}$ *leu1-32 ura4-D18*
FL875	$h^{?}$ *nda3-KM311 ndc80::ndc80-GFP-HA-nat*
FL877	$h^{?}$ *nda3-KM311 ndc80::ndc80-GFP-HA-nat cnp20:: cnp20-14A-TAP-kanMX6*
FL878	$h^{?}$ *nda3-KM311 ndc80::ndc80-GFP-HA-nat cnp20::cnp20-TAP-kanMX6*
FL882	$h^{?}$ *cnp1-1 ccp1::GFP-ccp1-kanMX6*
FL883	h^{-} *cnp20:: cnp20-9-GFP-kan leu1-32 ade6-M210 ura4-D18 his3-D1*
FL885	$h^{?}$ *cnp1-1 ccp1::GFP-ccp1-kan cnp20::cnp20-mCherry-kan*
FL886	$h^{?}$ *cnp20Δ1-55 ade6*$^{+}$*::cnp1-GFP leu1-32 ura4-D18 his3-D1*
FL887	$h^{?}$ *mis16-53 cnp20::cnp20-GFP-kan leu1-32*
FL888	$h^{?}$ pREP1-*cnp1-mCherry cnp20-9-ura4*$^{+}$*-his5*$^{+}$ *ccp1::GFP-ccp1-kanMX6 leu1-32 ade6-M210 ura4-D18*
FL889	$h^{?}$ *cnp20-9-ura4*$^{+}$*-his5*$^{+}$ *ndc80::ndc80-GFP-HA-kan leu1-32 ura4-D18*
FL900	$h^{?}$ *nda3-KM311 ndc80::ndc80-GFP-HA-nat cnp20:: cnp20-14D-TAP-kan*
FL901	$h^{?}$ *cnp20-9-ura4*$^{+}$*-his5*$^{+}$ *ccp1::GFP-ccp1-kanMX6 Ish1-mCherry-hph leu1-32 ade6-M210 ura4-D18*
FL902	$h^{?}$ *cnp1-1 cnp20::cnp20-GFP-kan*
FL903	$h^{?}$ *cnp20-9-ura4*$^{+}$*-his5*$^{+}$ *ade6*$^{+}$*::cnp1-GFP leu1-32 ura4-D18*
FL904	$h^{?}$ *ccp1::GFP-ccp1- ura4*$^{+}$ *cnp20::cnp20-mCherry-kanMX6 leu1-32 ade6-M210 ura4-D18*
FL905	$h^{?}$ *ccp1::GFP-ccp1- ura4*$^{+}$ *cnp20::cnp20-14A-mCherry-kanMX6 leu1-32 ade6-M210 ura4-D18*
FL906	$h^{?}$ pREP2-*sad1-mCherry ccp1::GFP-ccp1-kanMX6 leu1-32 ade6-M210 ura4-D18*
FL907	$h^{?}$ pREP2-*sad1-mCherry cnp20-14D ccp1::GFP-ccp1-kanMX6 leu1-32 ade6-M210 ura4-D18*
FL908	$h^{?}$ *cnp20::cnp20-TAP-kan ccp1:: GFP-ccp1-nat leu1-32 ade6-M210 ura4-D18*

Supporting References:

1. Tang X*, et al.* (2011) Marker reconstitution mutagenesis: a simple and efficient reverse genetic approach. *Yeast* 28(3):205-212.
2. Zhao Y & Boeke JD (2018) Construction of Designer Selectable Marker Deletions with a CRISPR-Cas9 Toolbox in Schizosaccharomyces pombe and New Design of Common Entry Vectors. *G3 (Bethesda)* 8(3):789-796.
3. Dong Q & Li F (2018) Antibody Pull-Down Experiments in Fission Yeast. *Methods Mol Biol* 1721:117-123.
4. Hagan IM, Grallert A, & Simanis V (2016) Synchronizing Progression of Schizosaccharomyces pombe Cells from Prophase through Mitosis and into S Phase with nda3-KM311 Arrest Release. *Cold Spring Harb Protoc* 2016(8).
5. Li F*, et al.* (2008) Lid2 Is Required for Coordinating H3K4 and H3K9 Methylation of Heterochromatin and Euchromatin. *Cell* 135(2):272-283.
6. Dong Q*, et al.* (2016) Ccp1 Homodimer Mediates Chromatin Integrity by Antagonizing CENP-A Loading. *Mol Cell* 64(1):79-91.
7. He H*, et al.* (2017) Coordinated regulation of heterochromatin inheritance by Dpb3-Dpb4 complex. *Proc Natl Acad Sci U S A* 114(47):12524-12529.
8. Yang J*, et al.* (2018) Heterochromatin and RNAi regulate centromeres by protecting CENP-A from ubiquitin-mediated degradation. *PLoS Genet* 14(8):e1007572.
9. McCloy RA*, et al.* (2014) Partial inhibition of Cdk1 in G 2 phase overrides the SAC and decouples mitotic events. *Cell Cycle* 13(9):1400-1412.
